# Anti-lung cancer therapy using nano-assembly particles of traditional Chinese Medicine formula

**DOI:** 10.1016/j.mtbio.2025.102502

**Published:** 2025-11-02

**Authors:** Yuanfeng Fu, Fei Xia, Lingling Sun, Minyi Guan, Qingchao Tu, Xiangjun Qi, Jigang Wang, Lizhu Lin, Chong Qiu

**Affiliations:** a School of the First Clinical Medicine, Guangzhou University of Chinese Medicine, Guangzhou, Guangdong, 510006, China; b State Key Laboratory for Quality Ensurance and Sustainable Use of Dao-di Herbs, Artemisinin Research Center, and Institute of Chinese Materia Medica, China Academy of Chinese Medical Sciences, Beijing, 100700, China; c The First Affiliated Hospital of Guangzhou University of Chinese Medicine, Guangzhou, Guangdong, 510006, China; d Guangdong Clinical Research Academy of Chinese Medicine, Guangzhou, Guangdong, 510006, China; e Institute of Acupuncture and Moxibustion, Shandong University of Traditional Chinese Medicine, 4655 University Road, Jinan 250355, China; f Department of Nephrology, Guangdong Provincial Clinical Research Center for Geriatrics, Shenzhen Clinical Research Center for Geriatric, Shenzhen People's Hospital, (The Second Clinical Medical College, Jinan University, The First Affiliated Hospital, Southern University of Science and Technology), Shenzhen, 518020, China

**Keywords:** Traditional Chinese Medicine, Nano-assembly particles, Lung cancer, Yi-Fei-San-Jie formula

## Abstract

Nano-assembly particles (NAPs) isolated from Traditional Chinese Medicine (TCM) formulations retain a better multi-component overall effect than isolated single active ingredient, which gradually emerging as a transformative approach for the pharmacodynamic and pharmacological research of TCM. This study introduced an innovative research paradigm for investigating the mechanisms of TCM formulas and proposes the groundbreaking concept of “TCM nano-formulated prescriptions” using Yi-Fei-San-Jie Formula (YFSJF) as a clinically study model. Firstly, based on the favorable outcomes in 31 clinical patients with pulmonary space-occupying lesions who received YFSJF treatment, the anti-tumor efficacy of NAPs isolated from the YFSJF decoction was systematically evaluated. Furthermore, the basic material components and underlying mechanism of NAPs were elucidated. Finally, 248 artificially synthetic nanoparticles developed by central bioactive components of YFSJF were used to study the self-assembly process, anti-tumor activity and safety profile. This study firstly demonstrated the potential NAPs-based replacement of traditional formulas and establishes a pioneering methodology for screening multi-component NAPs.

## Highlights

• Discovers NAPs in YFSJF decoction as primary anti-tumor components.
• Proposes “TCM nano-formulated prescriptions” via novel NAPs research paradigm.
• Shows extracted YFSJF NAPs match crude formula efficacy in vivo.
• Elucidates NAPs mechanism via integrated transcriptomics & mass spectrometry.
• Validates synthetic NAPs replace traditional formula, enabling new screening.

## Graphical abstract

**Figure:**
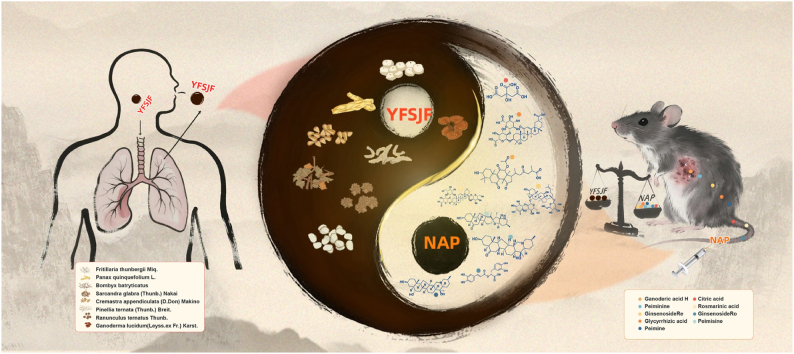

## 1 Introduction

Lung cancer, characterized by multiple gene mutations and its refractory nature, poses significant challenges in achieving satisfactory clinical outcomes with a single drug or treatment modality, resulting in 18 million deaths annually [1]. Therefore, there is an urgent necessity for the development of safe and effective novel treatment strategies. Yi-Fei-San-Jie Formula (YFSJF), a Traditional Chinese Medicine (TCM) formula originally termed the Yiqi Chutan formula, has been used as an adjuvant therapy for lung cancer since 2005. Clinical studies have demonstrated survival benefits in YFSJF-treated patients [2], while preclinical models demonstrate both toxicity reduction and efficacy enhancement [[3], [4], [5]]. However, the active components and the mechanistic basis of YFSJF remain incompletely characterized, restricting its further clinical development and application.

TCM formulations have long been the cornerstone of herbal therapeutics, with the complex chemical systems formed during decoction considered the primary material basis of pharmacological effects. However, conventional research focusing on isolated chemical components has overlooked the holistic nature of these formulations, significantly limiting their broader application. Emerging evidence has identified naturally occurring nano-assembly particles (NAPs) in extracts or decoctions of hundreds of herbs, including *Coptis chinensis* [6] and *Hypericum perforatum* [7,8], suggesting that NAPs may constitute a fundamental material basis for TCM efficacy. Recent studies demonstrate that NAPs derived from TCM formulations retain multifunctional therapeutic effects, exhibiting significant bioactivities such as anti-inflammatory and antimicrobial properties. For example, NAPs of Baihu Tang and Gegen Qinlian Tang modulate inflammatory pathways (e.g., NF-κB and MAPK), effectively suppressing cytokine release and alleviating inflammation [9]. NAPs of compound TCM QY305 mitigate epidermal growth factor receptor inhibitor-induced dermatologic adverse events and diarrhea through neutrophil recruitment regulation [10].

The paradoxical combination of potent bioactivity and compositional complexity in NAPs has spurred the development of artificial self-assembled nanoparticles using identified active components. These engineered systems offer distinct advantages, including defined composition, structural clarity, flexible combinatorial possibilities, and simplified preparation. Representative examples include berberine-based nanoparticles (NPs) assembled with rhein, cinnamic acid, tannic acid, or chlorogenic acid demonstrating enhanced anti-inflammatory effects [[11], [12], [13]]; gallic acid-derived polyphenol hydrogels inhibiting *Escherichia coli* and *Staphylococcus aureus* [14]; celastrol/erianin assemblies exhibiting improved tumor targeting and enhanced permeability and retention effects in breast cancer therapy [15]. Nevertheless, such reductionist approaches, divorced from their original formulations, fail to systematically elucidate NAPs mechanisms or recapitulate the multi-component, multi-target paradigm characteristic of TCM formulations. Therefore, comprehensive mechanistic investigations of formulation-derived NAPs remain imperative.

**Scheme 1:**
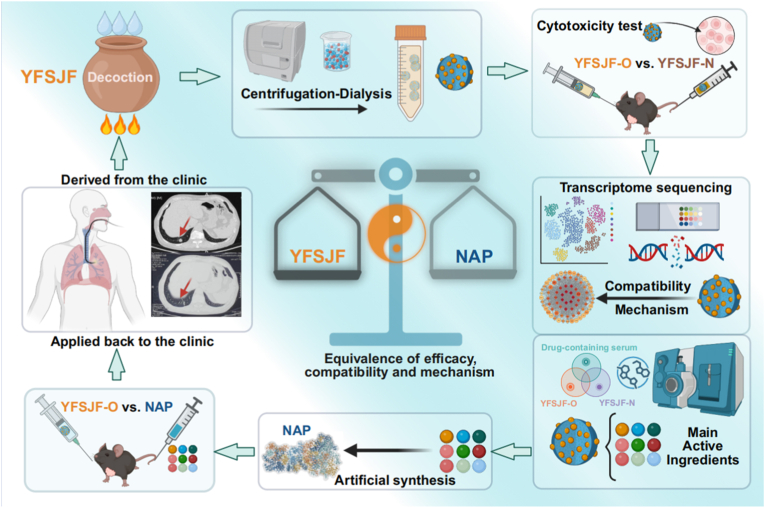
Summary of the study on the equivalence of YFSJF to NAPs.

Based on evidence of naturally occurring NAPs in herbal formulations, this study developed a novel research strategy to investigate formulation mechanisms through comparative validation of extracted NAPs and synthetic assemblies against original formulations. Using YFSJF as a model, this approach including (Scheme 1): (1) Validated the presence of NAPs in YFSJF decoction via dynamic light scattering (DLS) and transmission electron microscopy (TEM), supporting the hypothesis of NAPs as primary active components; (2) Demonstrated comparable anti-tumor efficacy between extracted NPs and crude formulation in animal models; (3) Elucidated pharmacodynamic compatibility mechanisms through integrated transcriptomics and mass spectrometry; (4) Synthesized bioinspired NAPs using predominant active components identified using mass spectrometry; (5) Established a structural rationale for multi-component NAPs through molecular dynamics (MD) simulations; (6) Confirmed formulation equivalence through tumor-bearing mouse studies. This systematic investigation firstly establishes a feasible framework for deciphering TCM formulation mechanisms through structural and functional analysis of multi-component NAPs, advancing both the understanding of herbal compatibility and the development of nano-formulated prescriptions.

## 2 Results

### 2.1 Clinical applications of YFSJF

**Fig. 1:**
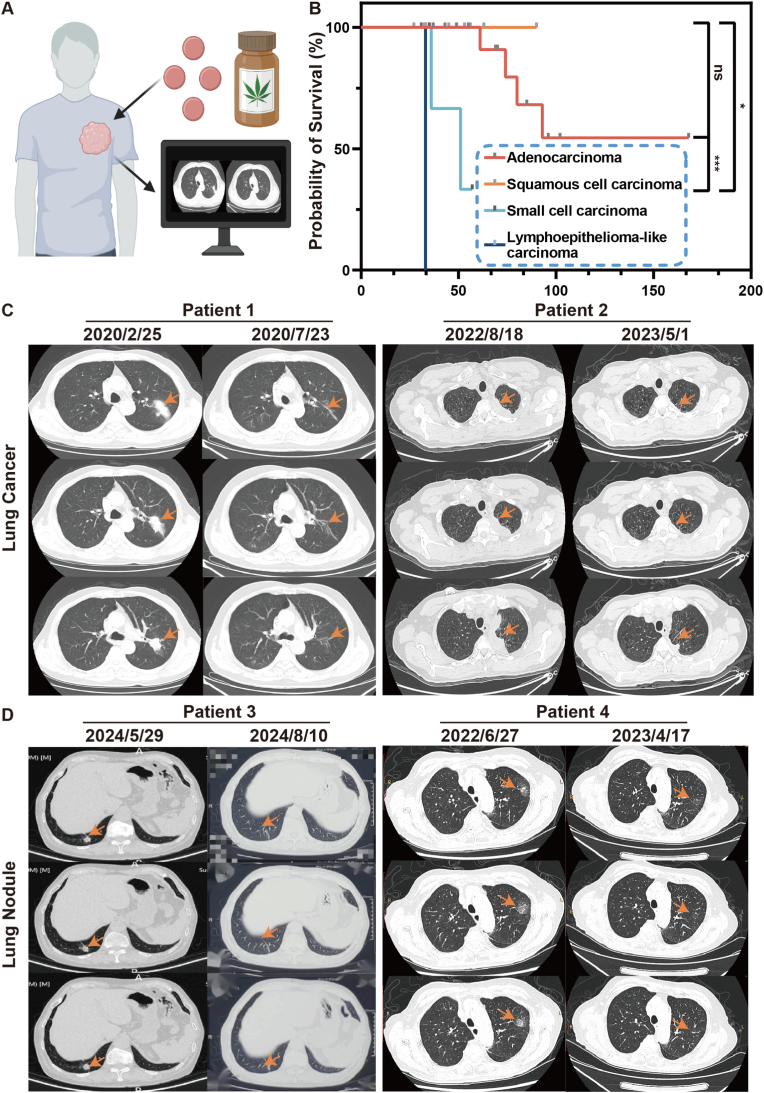
Clinical data results. (A) Schematic diagram of the clinical drug evaluation of YFSJF. Lung computed tomography (CT) scans of patients who received YFSJF to evaluate its treatment effect on lung lesions. (B) Survival curves for 31 patients, divided by pathological type. (C) Comparison of CT efficacy evaluation of patients with lung cancer. (D) Comparison of CT efficacy evaluation of patients with lung nodules. The arrows indicate the location of the lesions. Statistical significance: ∗p < 0.05 and ∗∗∗p < 0.001.

The therapeutic potential of TCM in the treatment of advanced lung cancer is garnering increasing attention. Previous studies have reported the synergistic effects of YFSJF when combined with conventional anticancer therapies. The survival of 31 patients with pathologically confirmed lung cancer who were admitted to the Cancer Center of the First Affiliated Hospital of Guangzhou University of Chinese Medicine was retrospectively analyzed (Table 1, Fig. 1A and B). The integration of YFSJF with standard treatment regimens demonstrated favorable clinical outcomes across both small cell lung carcinoma and adenocarcinoma subtypes, with a median overall survival exceeding 5 years. Among confirmed cases of lung cancer (Fig. 1C): Patient 1 was a 71-year-old male diagnosed with moderately-to-poorly differentiated squamous cell carcinoma in the left upper lobe who achieved partial response (PR) after 5 months of YFSJF therapy. The tumor mass changed from 5.2 cm × 2.8 cm to a linear opacity. Patient 2 was a 66-year-old male with small cell-type neuroendocrine carcinoma who maintained PR status following 8 months of YFSJF treatment, with no disease progression observed over a 3-year follow-up. The tumor size changed from 8.6 cm × 2.9 cm–2.4 cm × 1.8 cm. YFSJF exhibited therapeutic efficacy in pulmonary nodule management (Fig. 1D): Patient 3 was a 74-year-old male who presented with slightly dense radioactive distribution in the posterior basal segment of the right lower lobe (approximately 1.3 × 1.4 cm in size). Following 2 months of YFSJF treatment, a follow-up computed tomography (CT) scan revealed complete resolution of the nodule. Patient 4 was an 84-year-old female with a persistent left upper lobe nodule (from 2.7 cm × 1.7 cm–1.4 cm × 0.8 cm in size) for 3 months who showed significant improvement after 6 months of YFSJF administration. These clinical observations underscore the therapeutic value of YFSJF in lung cancer management, warranting further investigation into its material basis and mechanistic underpinnings. (Additional patient data available in Table S1).

**Table 1:** Baseline characteristics of the patients. The clinical stage of lung cancer is classified according to the Eighth edition of the TNM (tumor node metastasis classification).

Characteristics	Category	Number/Average
Gender	Male	25
	Female	6
Age (year)		64.4 ± 9.1
Pathological diagnosis	Adenocarcinoma	15
	Squamous cell carcinoma	9
	Small cell carcinoma	6
	Lymphoepithelioma-like carcinoma	1
Clinical stage	Ⅱ/B	1
	Ⅲ/A	4
	Ⅲ/B	5
	Ⅲ/C	2
	Ⅳ/A	11
	Ⅳ/B	7
Overall Survival (month)		63.3 ± 35.3

### 2.2 Characterization and bioequivalence validation of YFSJF NPs

**Fig. 2:**
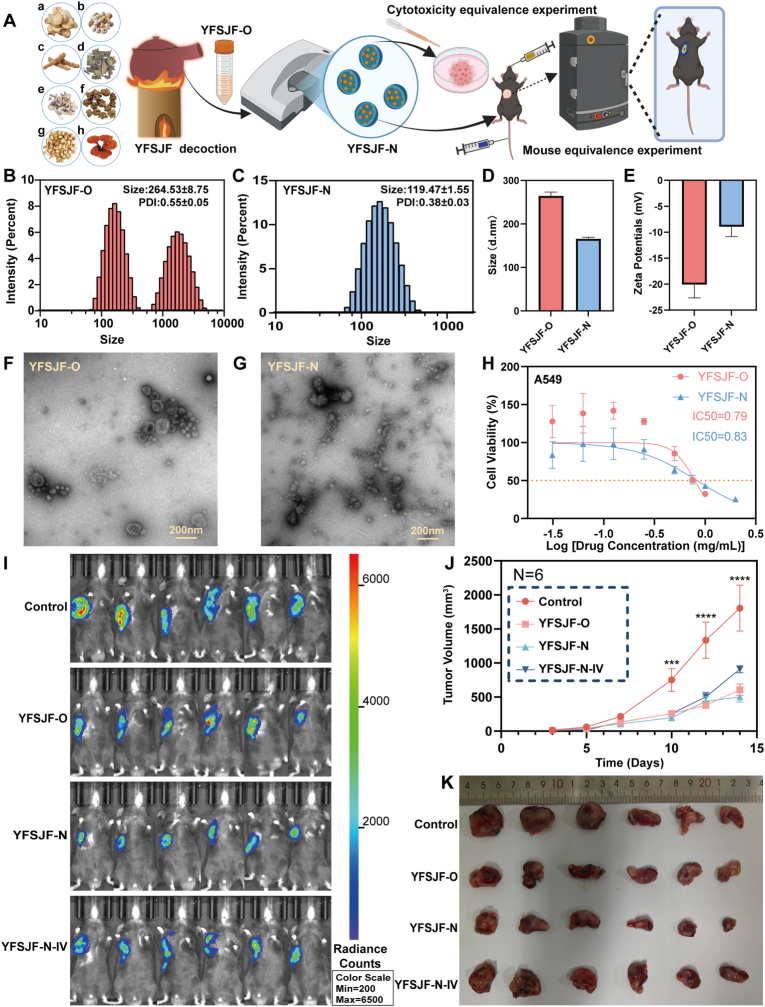
Characterization and equivalent verification of YFSJF. (A) Schematic diagram of drug extraction and equivalent verification. A. *Fritillaria thunbergii Miq.*, b. *Pinellia ternate (Thunb.) Breit.*, c. *Panax quinquefolium* L., d. *Sarcandra glabra (Thunb.) Nakai*, e *Bombyx batryticatus*, f. *Ranunculus ternatus Thunb.*, g. *Cremastra appendiculata (D.Don) Makino*, and h. *Ganoderma lucidum (Leyss.ex Fr.) Karst.* (B–C) Hydrodynamic size distributions of YFSJF-O and YFSJF-N. (D) Comparison of hydrodynamic size distributions of YFSJF-O and YFSJF-N. (E) Zeta potential of YFSJF-O and YFSJF-N. (F–G) TEM images of YFSJF-O and YFSJF-N. (H) Cytotoxicity experiment of YFSJF-O and YFSJF-N. (I) Fluorescence intensity of C57 BL/6J tumor-bearing mice (n = 6). The control group received NS, while YFSJF-O and YFSJF-N received the corresponding drug via gavage (500 μL/time), and YFSJF-N-IV received 200 μL/time via the tail vein. (J) C57 BL/6J tumor-bearing mice tumor volume growth curve. (K) C57 BL/6J tumor-bearing mice tumor measurement diagram. Statistical significance: ∗∗∗*p* < 0.001 and ∗∗∗∗*p* < 0.0001.

The YFSJF original solution (YFSJF-O) was prepared by boiling eight medicinal components (*Fritillaria thunbergii* Miq. 10 g, *Ranunculus ternatus* Thunb. 30 g, *Bombyx batryticatus* 10 g, *Sarcandra glabra* (Thunb.) Nakai 15 g, *Cremastra appendiculata* (D.Don) Makino 9 g, *Pinellia ternata* (Thunb.) Breit. 9 g, *Ganoderma lucidum* (Leyss.ex Fr.) Karst. 12 g, and *Panax quinquefolium* L. 6 g) in water. The NP-enriched fraction (YFSJF-N) was obtained by subjecting YFSJF-O to gradient centrifugation (10,000 rpm, 30 min) followed by 24 h dialysis. Comparative evaluation of YFSJF-O and YFSJF-N demonstrated comparable anti-tumor efficacy at both cellular and animal levels. YFSJF-O exhibits a polydisperse size distribution with multiple peaks, including a population with a mean hydrodynamic diameter of approximately 264 nm (polydispersity index [PDI] 0.55, Zeta potential −20 mV). DLS analysis revealed that YFSJF-N showed a moderately polydisperse distribution (119 nm, PDI 0.38) (Fig. 2A). TEM confirmed vesicular nanostructures in both preparations, verifying natural NAP formation (Fig. 2B–E). We observed the particle size changes of YFSJF by gradient centrifugation (Fig. S1A–D) and investigated the medium and dilution stability of YFSJF-N (Fig. S1E–F). Using Size Exclusion Chromatography - Multi-Angle Light Scattering (SEC-MALS) to detect YFSJF-O and YFSJF-N, we found that the peak of YFSJF-N was similar to that of YFSJF-O (Fig. S1G–H). *In vitro* testing yielded similar half-maximal inhibitory concentration (IC_50_) values against A549 cells (0.79 mg/mL for YFSJF-O vs. 0.83 mg/mL for YFSJF-N) (Fig. 2H), with YFSJF-N demonstrating potent activity against Lewis cells (IC_50_ 0.81 mg/mL) (Fig. S2). In C57BL/6J tumor-bearing mice, both oral and intravenous administration of YFSJF-N significantly reduced tumor fluorescence intensity (Fig. 2I) and volume, with oral delivery showing superior efficacy while maintaining comparable effects between YFSJF-N and YFSJF-O (Fig. 2J and K). Safety assessments revealed no histopathological abnormalities in major organs (Fig. S3A) or hematological chemistry parameters (Fig. S3B) following either administration route, confirming excellent biocompatibility.

### 2.3 Transcriptome sequencing and immunofluorescence analysis

**Fig. 3:**
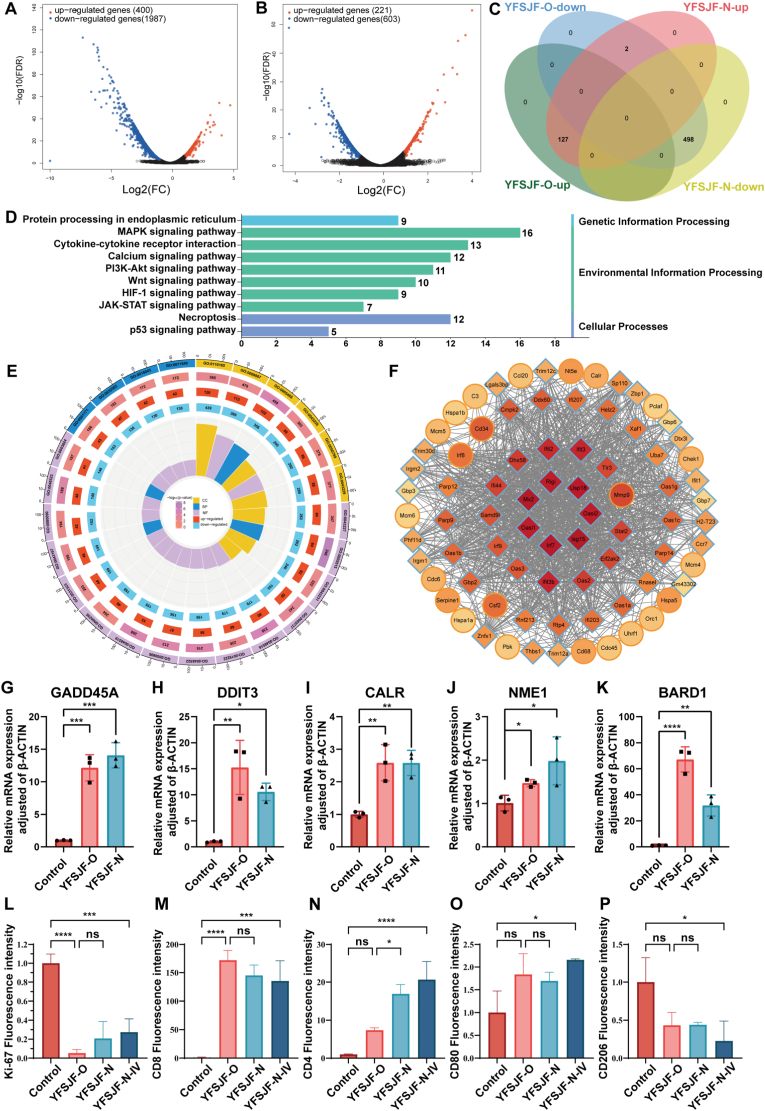
Transcriptome sequencing and immunofluorescence analysis. (A) Volcano plot of differentially expressed genes (DEGs) in YFSJF original (YFSJF-O) versus control. (B) Volcano plot of DEGs in NP-enriched fraction (YFSJF-N) versus control. (C) Venn diagram of co-expressed genes between YFSJF-O and YFSJF-N. (D) Kyoto Encyclopedia of Genes & Genomes (KEGG) pathway enrichment analysis showing the 10 significantly enriched pathways. (E) Gene ontology (GO) enrichment analysis of co-expressed genes. (F) Protein-protein interaction (PPI) network of key regulatory genes. (G–K) qPCR analysis of GADD45A, DDIT3, CALR, NME1, and BARD1 expression in Lewis cells treated with YFSJF-O or YFSJF-N. (L–P) Immunofluorescence staining and quantification of tumor tissues from Control, YFSJF-O, YFSJF-N, and YFSJF-N-IV groups, targeting Ki-67, CD8, CD4, F4/80+CD80, and F4/80+CD206. Statistical significance: ∗*p* < 0.05, ∗∗*p* < 0.01, ∗∗∗*p* < 0.001, ∗∗∗∗*p* < 0.0001.

To elucidate the mechanistic basis of YFSJF’s therapeutic effects, transcriptome profiling was performed on Lewis cells following treatment with either YFSJF-O or YFSJF-N. Comparative analysis revealed 2387 differentially expressed genes (DEGs) in YFSJF-O-treated cells versus controls, while YFSJF-N treatment resulted in 824 DEGs, with the top 100 most significant alterations visualized using heatmap analysis (Fig. S4). Intersection analysis identified 627 co-regulated DEGs that were subsequently subjected to gene ontology and Kyoto Encyclopedia of Genes and Genomes (KEGG) pathway enrichment (Fig. 3A–C). Both formulations exhibited similar regulatory patterns in shared gene networks, with KEGG analysis highlighting modulation of key pathways, including metabolic processes, MAPK signaling, and the PI3K-Akt cascade (Fig. 3D). The top 25 enriched terms across molecular functions, biological processes, and cellular components were systematically characterized (Fig. 3E and Table S2), while protein–protein interaction network analysis revealed central hub genes (Fig. 3F). To further validate the transcriptome findings, the expression of five representative genes (GADD45A, DDIT3, CALR, NME1, and BARD1) was examined by qPCR in Lewis cells following treatment with YFSJF-O or YFSJF-N. The results showed consistent expression patterns between the two groups, supporting the transcriptomic data and indicating that YFSJF-N reproduces the key molecular regulatory effects of YFSJF-O (Fig. 3G–K). Complementary immunofluorescence staining of tumor tissues revealed significant downregulation of the Ki-67 proliferation marker across treatment groups (Fig. 3L & Fig. S5), accompanied by enhanced CD4^+^ and CD8^+^ T cell infiltration (Fig. 3M and N). Furthermore, polarization analysis of tumor-associated macrophages showed increased F4/80+CD80^+^ (M1 phenotype) and decreased F4/80+CD206+ (M2 phenotype) populations (Fig. 3O and P), indicating YFSJF-mediated immunomodulation through both adaptive T-cell activation and innate macrophage reprogramming, thereby enhancing anti-tumor immunity.

### 2.4 Mass spectrometry-based identification of bioactive components

**Fig. 4:**
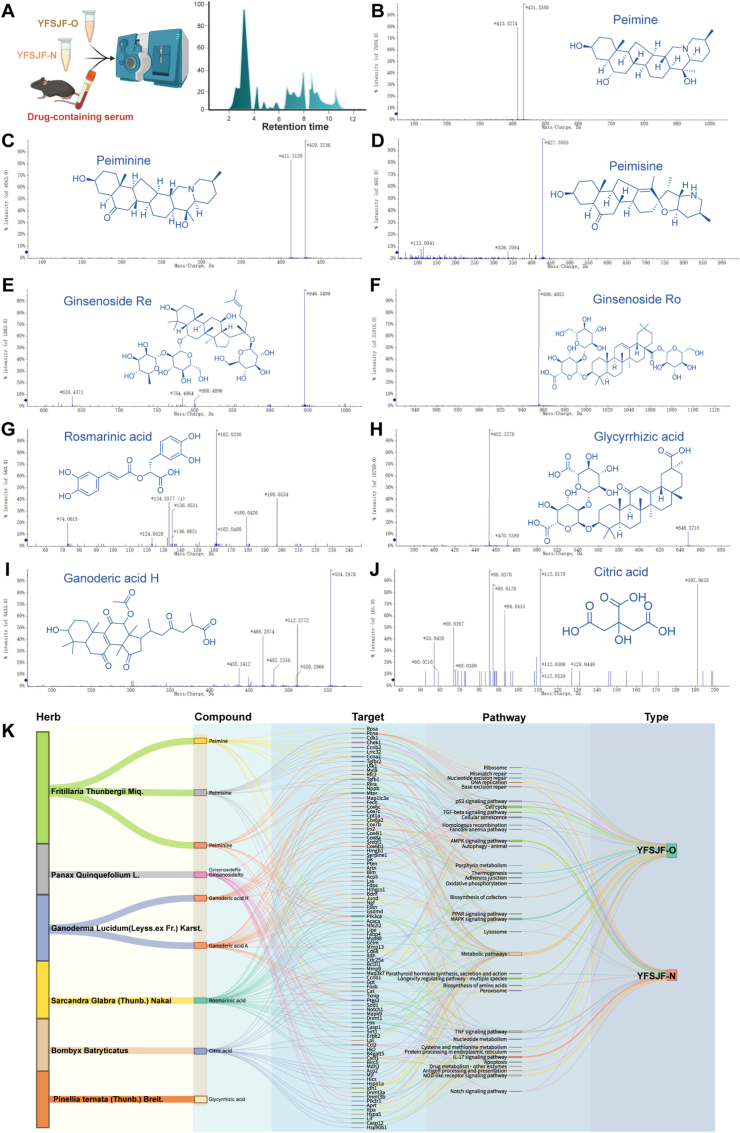
Mass spectrometry data results and association analysis. (A) Schematic diagram of high-pressure liquid chromatography (HPLC) detection samples, including lyophilized powder of YFSJF original (YFSJF-O) and NP-enriched fraction (YFSJF-N) solutions; 2 h drug-containing serum of YFSJF-O and YFSJF-N was gavaged to C57/BL 6J mice, and drug-containing serum of YFSJF-N was injected into the tail vein of C57/BL 6J mice. (B–J) Secondary ion peak diagrams of nine monomers and corresponding chemical structure diagrams. (K) Sankey diagram showing the association analysis from traditional Chinese medicine-compound-target-pathway.

Lyophilized YFSJF-O and YFSJF-N formulations were characterized by ultra-high performance liquid chromatography-mass spectrometry (UHPLC-Q-TOF-MS/MS), the composition of YFSJF NPs was analyzed, and the composition of drug-containing serum was analyzed in C57BL/6J mice that received oral gavage (YFSJF-O/YFSJF-N) or intravenous administration (YFSJF-N). Serum samples collected via orbital puncture at 2 h post-administration were similarly analyzed (Fig. 4A). Cross-referencing with known bioactive constituents from component herbs (Table S3) identified nine serum-detectable compounds: peimine, peiminine, peimisine, ginsenoside Re, ginsenoside Ro, rosmarinic acid, glycyrrhizic acid, ganoderic acid H, and citric acid (Fig. 4B–J). Transcriptome-guided network pharmacology modeling established compound-target-pathway relationships for these components, with ganoderic acid A substituted for the less-characterized ganoderic acid H to enhance predictive validity (Fig. 4K). The consistent detection of glycyrrhizic acid in both YFSJF-N and serum samples, combined with its established role as a nanocarrier with intrinsic anti-tumor properties [16,17], warranted its inclusion in subsequent NP synthesis.

### 2.5 Molecular dynamics simulation of self-assembly behavior

**Fig. 5:**
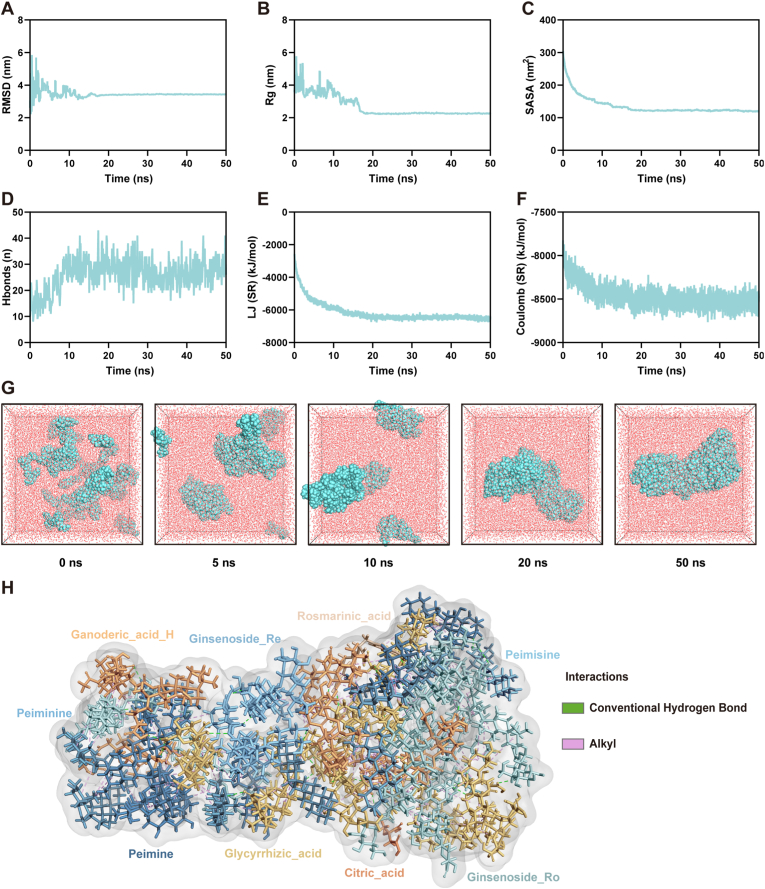
Molecular dynamics (MD) fitting. (A) System stability is characterized by root mean square deviation (RMSD). (B) Radius of gyration (Rg). (C) Solvent-accessible surface area (SASA). (D) Number of hydrogen bonds and interaction energy. (E) Changes in Lennard−Jones short-range interaction energy with simulation time. (F) Changes in Coulomb short-range interaction energy with simulation time. (G) Representative conformations at 0, 5, 10, 20, and 50 ns. (H) Cluster analysis of MD trajectories after 20 ns, showing the characteristic structure of clusters.

MD simulations were performed to validate the self-assembly capability of the nine identified bioactive components into stable NPs. The simulation system, with the molecular composition outlined in the Methods section, was run for 50 ns. Atomic root-mean-square deviation analysis revealed system equilibration at approximately 20 ns (Fig. 5A). Structural compactness was evaluated using the radius of gyration and solvent-accessible surface area, with both showing significant decreases and subsequent stabilization during the simulation (Fig. 5B and C), confirming the spontaneous formation of tightly packed nanostructures. Hydrogen bond analysis demonstrated increasing intermolecular interactions during assembly (Fig. 5D), while energy decomposition revealed dominant contributions from Lennard−Jones short-range interactions over Coulombic forces (Fig. 5E and F, Table S4), indicating van der Waals forces as the primary driving mechanism. Representative snapshots at different time points (Fig. 5G) illustrated the dynamic transition from disordered molecules to stable clusters. Cluster analysis of the final 20 ns trajectory identified characteristic assembled structures stabilized primarily through hydrogen bonding and alkyl interactions (Fig. 5H).

### 2.6 Screening of artificially synthesized NAPs

**Fig. 6:**
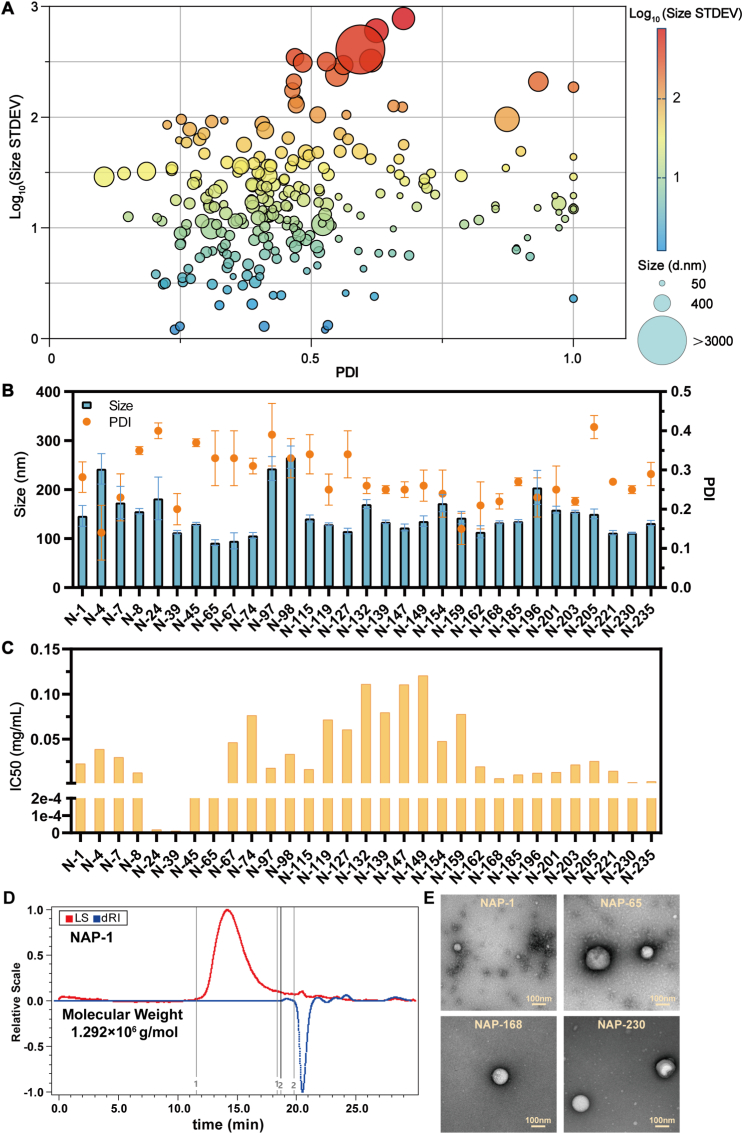
Artificial synthesis and composition screening. Bubble plot displaying particle size distribution of 248 combinatorial formulations. (B) Dynamic light scattering (DLS) characterization results for 31 selected NAPs. (C) Cytotoxicity profiles of 31 NAP preparations in Lewis cells. (D) SEC-MALS determination of N-1, showing an average molecular weight of 1292 × 10^6^ g/mol. (E) TEM images of representative NAP formulations (1, 65, 168, 230).

Based on the mass spectrometry results of drug-containing serum and MD simulations, we hypothesized that the relative abundance of components in vivo could inform the assembly of bio-inspired NPs. Therefore, as an initial proof-of-concept, the nine bioactive monomers were mixed at ratios reflecting their relative MS response values in the serum (Table S5), aiming to approximate their potential pharmacological contribution. The monomers, individually dissolved in anhydrous ethanol, were combined accordingly and evaporated in a 25 mL round-bottom flask at 37 °C. Subsequent reconstitution with deionized water yielded self-assembled NP solutions (N-1), exhibiting a hydrodynamic diameter of 126.83 ± 1.21 nm with a PDI of 0.24. To validate the assembly hypothesis, 248 distinct NAP combinations were prepared and characterized using DLS (Fig. 6A and B, Table S7), from which 31 formulations with diameters <200 nm and PDI <0.4 were selected for preliminary cytotoxicity screening (Fig. 6C, Table S8). Molecular mass determination via DLS revealed that N-1 complexes averaged 1291.06 kDa (Fig. 6D), with estimated stoichiometric ratios of constituent molecules as follows: peimine (631 molecules, 21.10 %), glycyrrhizic acid (315 molecules, 20.08 %), and ginsenoside Ro (252 molecules, 18.68 %) are the major components, together accounting for nearly 60 % of the total mass. Secondary components include ganoderic acid H (378 molecules, 15.13 %), peiminine (441 molecules, 14.67 %), and ginsenoside Re (63 molecules, 4.62 %). Minor constituents are peimisine (63 molecules, 2.09 %), citric acid (126 molecules, 1.87 %), and rosmarinic acid (63 molecules, 1.76 %) (Table S6). Four representative formulations—N-1 (all nine components), N-65 (peimine/ginsenoside Re/ginsenoside Ro/glycyrrhizic acid/ganoderic acid H/citric acid), N-168 (peimisine/ginsenoside Re/ginsenoside Ro/rosmarinic acid), and N-230 (peimisine/ginsenoside Ro/rosmarinic acid)—exhibited uniform spherical nanostructures under TEM (Fig. 6E).

### 2.7 Therapeutic evaluation of NAPs in Lewis tumor-bearing mice

**Fig. 7:**
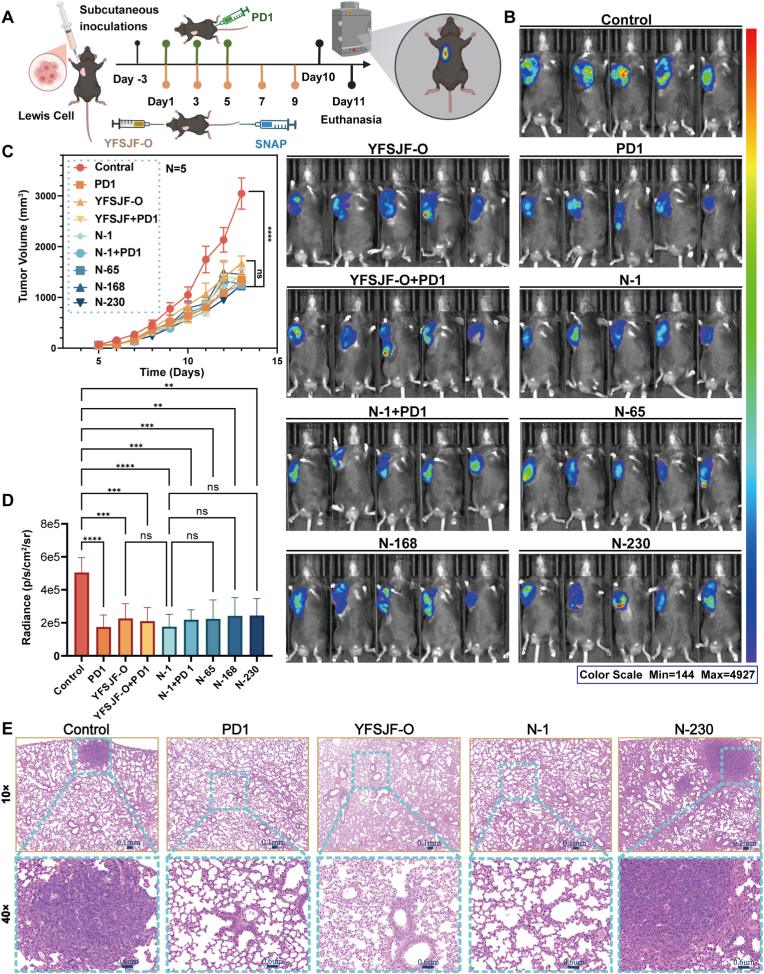
Equivalence verification of synthetic nanoparticles. (A) Schematic diagram of mouse experiment. Nine groups of C57 BL/6J mice (n = 5) were subcutaneously inoculated with Lewis cells, namely control, YFSJF original (YFSJF-O), PD1, YFSJF-O + PD1, N-1, N-1+PD1, N-65, N-168, and N-230. YFSJF-O was orally administered once every 2 days, PD-1 was intraperitoneally injected three times, 200 μg/time, and synthetic NAP was administered via the tail vein once every 2 days, 10 mg/kg. (B) Fluorescence intensity of tumor-bearing mice. (C) Mouse tumor growth curve. (D) Statistical graph of mouse tumor fluorescence intensity. (E) Hematoxylin & eosin (H&E) staining of the lung in mice with various treatments. Statistical significance: ∗∗*p* < 0.01, ∗∗∗*p* < 0.001, and ∗∗∗∗*p* < 0.0001.

Following initial synthesis and characterization, the anti-tumor efficacy of artificial NAPs was evaluated in C57BL/6J mice. To directly assess the intrinsic bioactivity of the synthetic particles and avoid confounding factors associated with oral absorption, NAPs were administered via the tail vein (Fig. 7A). Tumor fluorescence imaging and volume measurement after five treatment cycles (Fig. 7B–D) revealed that all treatment groups, including PD1 monotherapy, YFSJF-O, N-1, and their combinations with PD1, showed significant tumor suppression compared to the control group. Notably, the efficacy of N-1 was comparable to that of the original formula YFSJF-O. However, the combination of either YFSJF-O or N-1 with PD1 did not lead to a significant improvement in tumor inhibition compared to PD1 treatment alone. Histopathological analysis identified pulmonary metastases in the control and N-230 groups, whereas N-1, YFSJF-O, and PD1-treated animals (including combination groups) showed no metastatic spread, indicating that the metastasis-inhibitory effect of N-1 and YFSJF-O was at least equivalent to that of PD1. Histopathological analysis identified pulmonary metastases in the control and N-230 groups, whereas N-1, YFSJF-O, and PD1-treated animals showed no metastatic spread, indicating the superior metastasis inhibition of N-1, matching the original formula. Comprehensive safety evaluation confirmed the absence of pathological alterations in major organs (Fig. S6) and normal hematological parameters (Fig. S7) across all NAP treatment groups.

## 3 Discussion

The traditional paradigm of drug discovery from TCM has predominantly focused on isolating active monomer molecules (AMMs), as exemplified by the successful development of artemisinin and camptothecin derivatives [18,19]. While this AMM-based approach has yielded clinically valuable therapeutics, it poses significant limitations when applied to most herbal medicines. The purification process inherently disrupts the natural molecular interactions among various chemical constituents, consequently altering their pharmacological activities, modes of action, and pharmacokinetic profiles. This reductionist strategy often yields isolated monomers that fail to replicate the therapeutic effects of the original herbal extracts, while mechanistic studies on single compounds cannot adequately explain the compatibility principles underlying formula composition [20].

TCM formulations contain diverse chemical components, including polysaccharides, glycosides, organic acids, alkaloids, sterols, triterpenes, and proteins. During decoction, these bioactive molecules readily interact to form NAPs through various molecular interactions such as acid–base complexation between alkaloids and glycosides/organic acids/tannins [21]. Substantial evidence demonstrates that NAPs, rather than free monomers, serve as the actual functional executors of pharmacological effects in herbal formulations [9,10,22]. This study focuses on YFSJF, a clinically validated TCM formulation with demonstrated therapeutic effects in qi tonification, phlegm resolution, and detoxification. Its therapeutic benefits in reversing immune escape [5] and overcoming epidermal growth factor receptor-tyrosine kinase inhibitor (EGFR-TKI) resistance [23] have been validated, with ongoing research elucidating its anti-tumor mechanisms [3,4]. Retrospective clinical data (Fig. 1) further support the clinical relevance of this investigation. Our study provides compelling evidence that NAPs constitute the primary effectors of herbal formula efficacy. The integration of mass spectrometry data with synthetic validation supports a contemporary reinterpretation of the traditional “Jun-Chen-Zuo-Shi” (King-Minister-Assistant-Courier) compatibility theory: NAPs represent the “Jun” component responsible for multi-target, multi-pathway regulation; free compounds such as polysaccharides serve as “Chen” with supplementary functions; assembly-facilitating compounds (proteins/peptides/amino acids) act as “Zuo”; while solvents function as “Shi” for delivery. Integrating with the molecular compatibility theory [24,25], this study introduces the innovative concept of “Nano-Formulated TCM Prescriptions”: systematically designed NP complexes that consistently reproduce the original formula efficacy through pharmaceutically engineered multi-component assemblies guided by traditional compatibility principles and modern analytical techniques.

Extensive research has established the anti-tumor properties of *F. thunbergii* and its principal bioactive alkaloids—peimine, peiminine, and peimisine [26,27]. These compounds demonstrate multifaceted regulatory effects on critical signaling pathways, including mitogen-activated protein kinase (MAPK), Signal transducer and activator of transcription 3 (STAT3), nuclear factor kappa-B (NF-κB), Wnt/β-catenin, and JAK/STAT, contributing to their ability to inhibit tumor migration and modulate immune responses through M2 macrophage polarization [28,29]. Peimisine has been shown to regulate the transforming growth factor-β (TGF-β) pathway to inhibit lung cancer cell metastasis and can downregulate the JAK/STAT pathway [30,31]. Similarly, ginsenosides exhibit broad-spectrum anti-tumor activities: Ginsenoside Re suppresses tumor proliferation through multiple mechanisms, including adenosine activated protein kinase α1 (AMPKα1)/Stimulator of Interferon Genes (STING) pathway inhibition and EMT blockade in non-small cell lung cancer [[32], [33], [34]], while regulating PI3K/Akt, MAPK, and Nrf2/HO-1 pathways [35]. Ginsenoside Ro can significantly inhibit the proliferation of liver cancer cells through the MAPK signaling pathway [36]. The results of drug-containing serum in this study indicate that ganoderic acid H can indeed remain in the body for a longer time, and pharmacokinetic studies have confirmed this effect [37]. The simultaneous upregulation of GADD45A, DDIT3, CALR, NME1, and BARD1 indicates that YFSJF activates multiple tumor-suppressive pathways, including the p53/MAPK axis, calcium signaling–mediated immunogenic cell death, and DNA repair regulation [[38], [39], [40]]. Collectively, these molecular changes suggest a multi-faceted mechanism by which YFSJF enhances apoptosis, strengthens genomic stability, promotes immune recognition, and suppresses metastatic capacity in Lewis lung carcinoma. Building upon the characteristic “multi-target, multi-pathway” therapeutic mechanism of traditional formulations, this study successfully reconstituted nine bioactive TCM monomers into novel NAPs that collectively exhibit anti-tumor and immunomodulatory activities. This innovative approach effectively addresses the longstanding challenges of undefined composition and unclear mechanisms associated with conventional formulas while faithfully replicating the therapeutic profile of YFSJF. The rationally designed multi-component NAPs maintain the holistic therapeutic advantages of the original prescription while achieving precise compositional control and mechanistic clarity.

The aqueous solubility challenges of Fritillaria alkaloids (peimine, peiminine, and peimisine), which typically require organic solvents for pharmacological evaluation [30,41], were effectively overcome in both the YFSJF-N and synthetic N-1 formulations, demonstrating the solubilizing capacity of companion components in the nano-assembly system. Ginsenoside Ro belongs to the oleanolic acid type-ginsenoside, a pentacyclic triterpenoid compound that self-assembles into vesicle-like structures [42], which was confirmed in this study (Fig. 2, Fig. 6D). In previous studies, glycyrrhizic acid was mostly administered orally or topically in the form of hydrogels [43,44], which is not completely consistent with the administration route of common TCM compound prescriptions. In this study, glycyrrhizic acid formed a good aqueous solution when combined with other substances, providing a basis for the intravenous administration of glycyrrhizic acid to enhance bioavailability and facilitate early prescription screening. MD simulations revealed that the structural stabilization mechanism of YFSJF-derived NAPs primarily relies on hydrogen bonding and alkyl interactions. The extensive hydrogen bond network contributes to its structural integrity and three-dimensional stability, while the hydrophobic alkyl groups, consisting solely of carbon and hydrogen chains, form the internal framework, collectively explaining the observed amphiphilic vesicular architecture with hydrophilic exterior and hydrophobic core (Fig. 6D) [20,45]. These findings provide substantial evidence that NAP characterization offers valuable insights into both the pharmacological efficacy and compatibility rationale underlying therapeutic effects of YFSJF. Notably, we observed a hormetic-like growth promotion at low concentrations of the crude decoction (YFSJF-O) in vitro (Fig. 2H), an effect not observed with the nano-formulation (YFSJF-N). This difference may stem from the altered bioavailability or composition of the nano-assembly. The transcriptional regulation of the HIF-1 signaling pathway by YFSJF (Fig. 3D), which can exhibit dual roles in cell fate, provides a potential mechanistic clue for this context-dependent response.

This study represents a paradigm shift from conventional single-component investigations to the innovative synthesis of multi-component nano-complexes that faithfully replicate the therapeutic profile of traditional herbal formulations while maintaining favorable safety characteristics—an approach reported for the first time in current research [21,46,47]. The successful assembly of pharmacologically active monomers from different herbal sources, guided by mass spectrometry analysis, demonstrates the technical feasibility of this strategy and its potential for modernizing traditional formulations into precisely designed nano-prescriptions. Most significantly, this work establishes a novel scientific framework for TCM research by introducing the groundbreaking concept of “TCM Nano-Formulated Prescriptions,” while concurrently validating the unique scientific value of nanoscale analysis in deciphering the mechanistic basis of herbal formula efficacy. The convergence of traditional pharmacological techniques with advanced nanotechnology opens new avenues for developing next-generation herbal medicines with defined composition and enhanced therapeutic precision.

Several limitations should be acknowledged in this investigation: (1) The clinical translatability requires further validation, as the current study primarily relied on cellular and animal models. While retrospective clinical data confirmed YFSJF’s efficacy, neither YFSJF-N injection nor the synthetic N-1 has undergone clinical evaluation for potency and safety [48]. (2) Mechanistic understanding remains incomplete due to compositional complexity. Although transcriptomics and mass spectrometry identified potential pathways, the precise spatial organization and functional interplay of multicomponent NAPs demand advanced analytical strategies. (3) Manufacturing processes need optimization, as the conventional self-assembly method for N-1 production lacks systematic evaluation of yield and batch-to-batch consistency, with scalability and quality control standards yet to be established. It should be noted that this self-assembly model, while functionally representative, does not fully capture the full supramolecular complexity of the native herbal decoction. (4) Long-term safety profiles are undetermined, as acute toxicity studies demonstrated favorable safety, but repeated-dose toxicity assessments are pending, particularly regarding immunomodulatory effects and chronic organ toxicity. (5) Although transcriptome sequencing of Lewis cells provided valuable mechanistic insights, cell-based omics approaches have inherent limitations, as they cannot fully reflect the complexity of the tumor microenvironment or immune regulation. Future studies will incorporate transcriptomic and multi-omics analyses of tumor tissues from animal models to achieve a more comprehensive understanding of the pharmacodynamic mechanisms of TCM formulas. (6) While this study demonstrated the potent efficacy of YFSJF-O and synthetic N-1, the combination with anti-PD1 immunotherapy did not show a significant advantage over PD1 monotherapy in the Lewis lung carcinoma model used. This indicates that the potential for synergy requires further investigation in different tumor models or with alternative treatment schedules [49]. (7) Oral administration of TCM formulas and nano-assemblies may have a beneficial or negative impact on therapeutic results via intestinal microbial metabolism, resulting in indirect pharmacologic effects [45]. In this study, synthesized NAPs were administered intravenously to better assess their direct anticancer effects. Further studies will include oral and other administration routes to have a better knowledge of the efficacy and safety characteristics of these nano-formulated systems. These limitations highlight critical directions for future research, including enhanced translational studies, deeper mechanistic exploration, process optimization, and rigorous quality control development to advance “TCM Nano-Formulated Prescriptions” toward clinical application.

## 4 Conclusions

This study established a comprehensive research paradigm for investigating traditional Chinese herbal formulas using YFSJF as a model. The investigation progressed systematically from clinical efficacy data to NP extraction and bioequivalence validation, culminating in the successful development of artificially synthesized multi-component NAPs that demonstrated therapeutic equivalence with the original formula. Key findings include: (1) The nano-extracted fraction (YFSJF-N) exhibited comparable anti-tumor activity to crude YFSJF decoction (YFSJF-O), confirming NPs as the primary active components; (2) Mass spectrometry analysis identified nine bioactive monomers (peimine, peiminine, peimisine, ginsenosides Re/Ro, rosmarinic acid, glycyrrhizic acid, ganoderic acid H, and citric acid) through systematic comparison of YFSJF-O, YFSJF-N, and drug-containing serum; (3) The artificially assembled NP N-1, containing these nine components, replicated both the anti-tumor efficacy and safety profile of the original formula in animal studies, while demonstrating the feasibility of ternary + multi-component NAPs. Distinct from previous nanomedicine approaches to TCM, this investigation pioneers a methodology that simultaneously deciphers formula compatibility mechanisms at the nanoscale and develops compositionally defined nano-formulations as potential replacements for traditional preparations. Collectively, this work establishes a groundbreaking research framework for developing “TCM Nano-Formulated Prescriptions” derived from clinically validated herbal formulas.

## CRediT authorship contribution statement

**Yuanfeng Fu:** Writing – original draft, Formal analysis, Data curation, Conceptualization. **Fei Xia:** Writing – original draft, Formal analysis. **Lingling Sun:** Formal analysis, Data curation. **Minyi Guan:** Data curation. **Qingchao Tu:** Formal analysis. **Xiangjun Qi:** Data curation. **Jigang Wang:** Writing – review & editing. **Lizhu Lin:** Writing – review & editing, Funding acquisition. **Chong Qiu:** Writing – review & editing, Writing – original draft, Conceptualization.

## Methods

All methods are available in Supporting Information.

## Ethical approval

All patient information was collected after approval by the Ethics Committee of the First Affiliated Hospital of Guangzhou University of Chinese Medicine (K-2024-188). All animal studies were approved by the Institutional Animal Care and Use Committee and Animal Ethics Committee of the Institute of Chinese Materia Medica (2024B221).

## Declaration of competing interest

The authors declare that they have no known competing financial interests or personal relationships that could have appeared to influence the work reported in this paper.

## Data Availability

Data will be made available on request.
