# Supplementary material for: Anti-lung cancer therapy using nano-assembly particles of traditional Chinese Medicine formula
Source: Mater Today Bio. 2025 Nov 2;35:102502. doi: 10.1016/j.mtbio.2025.102502 (PMC12661425; doi:10.1016/j.mtbio.2025.102502)
Supplement: Multimedia component 1 [file mmc1.docx]

Supporting Information

**Materials and Method**

**Materials and reagents**

MD44-3.5KD dialysis bags were purchased from Beijing Weikehang Technology Co., Ltd. (Beijing, China). Dulbecco’s Modified Eagle Medium (DMEM) was purchased from ThermoFisher Scientific Inc (Beijing, China); phosphate buffered saline (PBS) (pH 7.4) was purchased from Service Biotechnology Ltd (Hong Kong, China). Sodium chloride hysiological solution (normal saline, NS) (H20083400) was purchased from Sichuan Kelun Pharmaceutical Co., Ltd. Peimine (BT-BM01), peiminine (BT-BM02), peimisine (BT-BM03), ginsenoside Re (BT-RS15), ginsenoside Ro (BT-RS24), rosmarinic acid (BT-DS13), ganoderic acid H (BT-LZ31) and Anti-mouse PD-1 (CD279)-In Vivo (Selleck, America). were purchased from Beijing BetterKang Biopharmaceutical Technology Co., Ltd. (Beijing, China). Primary antibodies Anti-Ki67 antibody (ab15580), Anti-CD80 antibody (ab254579), Anti-F4/80 antibody (ab300421), CD206/MRC1 (E6T5J) XP^®^ Rabbit mAb (CST 24595S)、CD4 (D7D2Z) Rabbit mAb (CST 25229S) and CD8α (D4W2Z) XP^®^ Rabbit mAb (CST 98941S) were purchased from Beijing BetterKang Biopharmaceutical Technology Co., Ltd. (Beijing, China).

**YFSJF preparation and characterization**

YFSJF (*Fritillaria thunbergii Miq.* 10g, *Ranunculus ternatus Thunb.* 30g, *Bombyx batryticatus* 10g, *Sarcandra glabra (Thunb.) Nakai* 15g, *Cremastra appendiculata (D.Don) Makino* 9g, *Pinellia ternate (Thunb.) Breit*. 9g, *Ganoderma lucidum (Leyss.ex Fr.) Karst.* 12g, *Panax quinquefolium L.* 6g) was added to 1000mL of cold water, soaked for 60min, boiled for 60min, and then the extract was filtered with gauze and allowed to cool to room temperature. The extract was centrifuged at 1000rpm and 4℃ for 30min using a high-speed centrifuge, the precipitate was discarded and the supernatant was collected to obtain YFSJF-O, which was stored at 4℃. The compound drug solution was centrifuged at 10000rpm at 4℃ for 30 min, then placed in a MD44-3.5KDa dialysis bag, dialyzed in double distilled water for 24 hours to obtain YFSJF-N, and stored at 4℃. Dynamic light scattering (Malvern zeta sizer, Malvern) was used to measure the size distribution and zeta potential of YFSJF-O and YFSJF-N. YFSJF-O and YFSJF-N solutions were diluted to 20% of their original concentrations. 10 μL of the diluted solution was applied to a carbon-coated copper grid and observed using a transmission electron microscope (TEM, Hitachi HT7700). The excess liquid around was absorbed by filter paper, and the sample was then immersed in lead citrate for staining. After staining for 2 min, the copper mesh was dried and transferred to the sample rod for observation under a transmission electron microscope.

**Cytotoxicity assay**

Inoculate 100 μL of 3×10^3^-7×10^3^ cells/well on a 96-well plate, add 100 μL of DMEM medium, and culture at 37°C, 5% CO_2_, and 90% humidity for 24 hours. On the second day, the cell growth status and density were observed under a microscope, and the wells with good growth status, uniform cell distribution and density were selected for the experiment. YFSJF-O and YFSJF-N solutions were added (the concentration gradient was 0.0625 mg/mL, 0.125 mg/mL, 0.25 mg/mL, 0.5 mg/mL, 0.75 mg/mL, 1 mg/mL, 1.5 mg/mL, and 2 mg/mL. The solution concentration was about 21 mg/mL for YFSJF-O and about 4.2 mg/mL for YFSJF-N in the form of freeze-dried solution). The cells were cultured at 37°C, 5% CO_2_, and 90% humidity for 72 hours, and the cells were tested at the time points. 10 μL of CCK-8 solution was added to each well. After incubation for 30 min, the absorbance was measured at 450 nm with an enzyme reader. Three to six replicate wells were assayed each time. The cytotoxicity of synthetic NAPs was investigated using the same methods as above.

**Animal experiments**

Male C57 BL/6J mice (5-6 weeks old, 18-21 g) were purchased from Vital River Laboratory Animal Technology Co. Ltd (Beijing, China) and used in accordance with the Animal Ethics and Welfare Committee of the China Academy of Traditional Chinese Medicine.

The mice were adaptively fed for 1 week, and the cells in the logarithmic growth phase were digested and decellularized with 0.25% trypsin, then centrifuged at 1000r/min for 3min with PBS or normal saline, washed twice, and trypsin and serum in the culture medium were washed away. The tumor was diluted with normal saline to a certain concentration of cell suspension, and 0.2 mL (containing 4×10^6^ cells) was inoculated at each inoculation point using a 1mL syringe. Lewis’s lung cancer cells were inoculated in the right axilla to establish a C57 BL/6 Lewis lung implant cancer model. On the second day of inoculation, mice were randomly divided into four groups, each consisting of 6 mice. The model group was given NS 0.5 mL The YFSJF-O and YFSJF-N groups were treated by gavage at a dose of 0.5 mL per mouse. The fourth group received an intravenous injection of YFSJF-N (YFSJF-N-IV) via the tail vein at a dose of 0.2 mL per mouse. All mice were administered medication every two days, and normal feeding was continued for 12 consecutive days. The body weight was weighed once every 3 days after inoculation. At the end of the experiment, 0.5-1 mL of blood was collected from the eyeballs of mice, and the mice were killed by cervical dislocation. Tumor tissue and lung tissue were collected and stored at -80°C for later use. The blood biochemistry of mice was tested, and HE staining of tumor, lung, liver and other tissues of mice was performed to verify the safety and effectiveness of YFSJF-N. The instrument for routine blood tests was an automatic blood analyzer (Shenzhen Mindray, BC-5180CRP), and the instrument for biochemical tests was a fully automatic biochemical analyzer (Toshiba, Japan, TBA40FR).

NAP animal equivalent experiment, tumor-bearing C57 BL/6J mice were modeled as before. We set up a total of 9 groups of mice, 5 mice in each group, namely Control, YFSJF-O, PD1, YFSJF-O+PD1, N-1, N-1+PD1, N-65, N-168, N-230. Among them, YFSJF-O was orally administered once every two days, 0.5 mL/time, PD-1 was intraperitoneally injected 3 times, and synthetic NAP was administered once every two days through the tail vein. According to the preliminary experiment to test the tolerance of mice, the final dosage of N-1 was 5 mg/kg, and the dosage of N-65, N-168, and N-230 was 10 mg/kg.

**RNA extraction for Transcriptome**

Lewis cells were seeded in 6 cm dishes at 1.5 million/dish, with 5 mL serum-free medium per dish, and YFSJF-O or YFSJF-N (concentration 0.8 mg/mL, 230 ul of drug solution added to 5.73 mL DMEM) drug-containing groups were placed in the same position. After 72 hours of action, the medium was aspirated, washed once with PBS, and 1.2 mL of TRIZoL/TRIeasy reagent was added to each dish. Lysed at 4°C (ice bath) for 30 min, and the cells were blown with a gun to completely lyse. The suspension was aspirated into a 1.5 mL centrifuge tube, stored at -80°C, and transported on dry ice. Then RNA quality was determined using 2100 Bioanalyser (Agilent) and quantified using the ND-2000 (NanoDrop Technologies). High-quality RNA sample (OD260/280=1.8~2.2, OD260/230≥2.0, RIN≥6.5, 28S:18S≥1.0, >10 μg) is used to construct sequencing library. ^[1]^

**Transcriptome analysis**

**Library preparation and** **sequencing**

RNA-seq transcriptome libraries were prepared following standard RNA library preparation procedure, using 1μg of total RNA. Shortly, messenger RNA was isolated with polyA selection by oligo(dT) beads and fragmented using fragmentation buffer. cDNA synthesis, end repair, A-base addition and ligation of the indexed adaptors were performed. Libraries were then size selected for cDNA target fragments of 200–300 bp on 2% Low Range Ultra Agarose followed by PCR amplified using Phusion DNA polymerase (NEB) for 15 PCR cycles. After quantified by TBS380, final libraries were sequenced using next generation sequencing platform (MGI-T7 or Illumina) (Shanghai BIOZERON Biotech. Co., Ltd) with PE150 mode according to the standard protocols.

| Step | Reagent Instrument | Company |
| --- | --- | --- |
| RNA extraction | TRIzol® Reagent | Invitrogen |
| Plant RNA extraction | Plant RNA Purification Reagent | Invitrogen |
| mRNA separation | magnetic grate | Invitrogen |
| Library preparation | VAHTS Universal V10 RNA-seq Library Prep Kit | MGI |
| quantify | TBS380 Picogreen | Invitrogen |
| Library amplification | cBot Truseq PE Cluster Kit v3-cBot-HS | MGI |
| Library recovery | Certified Low Range Ultra Agarose | Bio-Rad |

## Reads quality control and mapping

The raw paired end reads were trimmed and quality controlled by Trimmomatic^[2]^ with parameters (SLIDINGWINDOW:4:15 MINLEN:75) (version 0.36 http://www.usadellab.org/cms/uploads/supplementary/Trimmomatic).In detail， (1) Remove adapter sequences from reads, and remove reads without insert fragments due to adapter self-connection or other reasons;(2) Trim the base with low quality (quality value less than 20) at the end of the sequence (3 'end). If there was still a base with quality value less than 10 in the remaining sequence, the whole sequence was removed, otherwise the sequence was retained.(3) The reads with N ratio more than 10% were removed;(4) The sequences less than 75bp in length after adapter and quality pruning were discarded.Then clean reads were separately aligned to reference genome with orientation mode using Hisat2^[3]^ (https://ccb.jhu.edu/software/hisat2/index.shtml) software. This software was used to map with default parameters. The quality assessment of these data were taken by qualimap_v2.2.1^[4]^ （<http://qualimap.bioinfo.cipf.es/>）. Clean reads were aligned to the ribosome database of this species using the alignment tool bowtie2^[5]^ to evaluate the proportion of rRNA in the sample.Use featurecount（http://subread.sourceforge.net/) to count each gene reads.

## Differential expression analysis and Functional enrichment

The transcriptome data analysis were operated by CFViSA^[6]^ plantform, which contained transcriptome analysis pipeline and nearly 80 analysis tools spanning simple sequence processing, visualization, and statistics available for transcriptiome data.

To identify DEGs (differential expression genes) between the two different samples, the expression level for each gene was calculated using the fragments per kilobase of exon per million mapped reads (FRKM^[7]^) method.R statistical package edgeR^[8]^ (Empirical analysis of Digital Gene Expression in R, [http://www.bioconductor.org/packages/release/bioc/html/edgeR.html/](http://cufflinks.cbcb.umd.edu/)) was used for differential expression analysis. The DEGs between two samples were selected using the following criteria: the logarithmic of fold change was greater than 2 and the false discovery rate (FDR) should be less than 0.05. To understand the functions of the differentially expressed genes, GO functional enrichment and KEGG pathway analysis were carried out by Goatools^[9]^ (<https://github.com/tanghaibao/Goatools> ) and KOBAS^[10]^ (<http://kobas.cbi.pku.edu.cn/kobas3>). DEGs were significantly enriched in GO^[11]^ terms and metabolic pathways when their Bonferroni-corrected P-value was less than 0.05.In this analysis, GO annotation results of differential genes were sorted out and statistically analyzed based on level2，the abbreviation of GO is Gene Ontology Gene Ontology (http://www.geneontology.org/). KEGG^[12]^ (Kyoto encyclopedia of genes and genomes) : the differential genes could be displayed on the KEGG pathway map, and the KEGG annotated pathway map of differential genes could be displayed.

## Gene Set Enrichment Analysis

GSEA(Gene Set Enrichment Analysis）（http://www.gsea-msigdb.org/gsea/index.jsp）were used a predefined set of genes, usually derived from functional annotations or the results of previous experiments, to rank genes according to their degree of differential expression in the two classes of samples, and then tests whether the prespecified set of genes is enriched at the top or bottom of the ranking table. Use MsigDB（http://software.broadinstitute.org/gsea/msigdb）to enrich gene set. GO was plotted by https://www.bioinformatics.com.cn (last accessed on 10 Dec 2024), an online platform for data analysis and visualization.^[13]^

## PPI

The interaction relationship in the STRING protein interaction database (http://string-db.org) was used to analyze the protein interaction network of differential genes. For each species included in the database, we extracted the differential gene sets from the database and used cytoscape to construct the interaction network. We first mapped the differentially expressed genes to the tring database proteins, and constructed the protein-protein interaction network using the protein-protein interaction relationships on the alignment. Based on the filtered mass spectrometry and transcriptome data, a relationship between traditional Chinese medicine, compound, target, and pathway is built, and the Sankey diagram is visualized using the R package networkD3.

**RNA extraction**

Total RNA was extracted using an RNA extraction kit (Vazyme, #RC102, Nanjing, China) according to the manufacturer’s instructions. Briefly, Lewis’s adherent cells cultured in 6-well plates were washed by removing the supernatant, followed by lysis with 500 μL Buffer CRL per well. The lysates were transferred to RNA Columns Ⅰ and centrifuged at 12,000 rpm for 30 s. After sequential washes with Buffer RWA and Buffer RW (both supplemented with absolute ethanol prior to use), the columns were dried by centrifugation and total RNA was eluted in 30 μL RNase-free ddH₂O. RNA concentration and purity were determined spectrophotometrically, and samples were stored at −80 °C until use.

**Reverse transcription**

For cDNA synthesis, 1 ng of total RNA was reverse-transcribed in a 20 μL reaction containing 1 μL Random Primer (N9), 10 μL 2×TS Reaction Mix, and RNase-free water. Reverse transcription was carried out at 25 °C for 10 min, 42 °C for 15 min, and 85 °C for 5 s. The resulting cDNA was stored at −20 °C for subsequent analysis.

**Quantitative real-time PCR (qPCR)**

qPCR was performed in a 20 μL reaction system containing 5 μL cDNA template, 0.4 μL each of forward and reverse primers, 10 μL SYBR Green Master Mix, and 4.2 μL RNase-free water. Each sample was analyzed in triplicate. Amplification was conducted under the following conditions: 94 °C for 30 s, followed by 45 cycles of 94 °C for 5 s and 60 °C for 30 s. A melting curve analysis was performed to confirm the specificity of amplification. Relative mRNA expression levels were calculated using the 2^−ΔΔCt^ method, with GAPDH (or other designated housekeeping gene) as the internal control.

| Name | Gene sequence |
| --- | --- |
| Gadd45a-Mus-F | CCGAAAGGATGGACACGGTG |
| Gadd45a-Mus-R | TTATCGGGGTCTACGTTGAGC |
| Ddit3-Mus-F | CTGGAAGCCTGGTATGAGGAT |
| Ddit3-Mus-R | CAGGGTCAAGAGTAGTGAAGGT |
| Calr-Mus-F | AAGATGCCCGATTTTACGCAC |
| Calr-Mus-R | CCCACAGTCGATATTCTGCTC |
| NME1-Mus-F | AGGAGCACTACACTGACCTGA |
| NME1-Mus-R | GGTTGGTCTCTCCAAGCATCA |
| BARD1-Mus-F-2 | CCCCACCTCTGCTGAACAAT |
| BARD1-Mus-R-2 | CTGCAGGCTTCATGCTTAATAGA |

**Immunofluorescence staining of tumor tissue**

Paraffin-embedded mouse tumor tissue was cut into 10μm slices and placed in an oven at 60℃ for 2 hours. The slices were taken out and immersed in xylene I and xylene II for 10 min each for dewaxing. After dewaxing, immerse the slices in anhydrous ethanol I, anhydrous ethanol II, 90% ethanol, 80% ethanol, and 70% ethanol for 1 minute each to rehydrate the slices. After taking out the slices from 70% ethanol, immerse them in sodium citrate antigen repair solution and heat them at high temperature for 30 min for antigen repair. After completion, wait for the slices to cool to room temperature. Then, add endogenous peroxidase scavenger to the slices, place them in a wet box, incubate them at room temperature in the dark for 20 min, and then wash the slides 3 times, following the same steps as before. Add goat serum for blocking and incubate them at room temperature in the dark for 1 hour.

Dilute CD8 with antibody diluent at 1:500 and add it to the slide, incubate in a humidified box in the dark for 1 hour, wash the slide 3 times after completion, and follow the same steps as before. Dilute AXB secondary antibody with antibody diluent at 1:3 and add it to the slide, incubate in a humidified box in the dark for 10 min at 37℃, wash the slide 3 times after completion, and follow the same steps as before. Dilute XTSA 480 dye with signal amplification solution at 1:100 and add it to the slide, incubate in a humidified box in the dark for 10 min at room temperature, wash the slide 3 times after completion, and follow the same steps as before.

Add DAPI to stain the cell nucleus, place in the dark for 10 min, soak the slice in PBS for 5 min after completion, and wash 3 times. Add sealing agent to seal the slide and let it dry. After sealing, the expression of each protein in the sample can be observed by the fluorescence scanning imaging system. The CD4 antibody diluent was diluted 1:100 and added onto the slide. The incubation was carried out in a humidified box away from light for 1 hour. After the incubation, the slide was washed 3 times. The rest of the steps were the same as before.

F4/80 was diluted 1:5000 with antibody diluent and added to the slide, incubated in a humidified box in the dark for overnight, and the slide was washed 3 times after completion. The steps were the same as before. AXB secondary antibody was diluted 1:3 with antibody diluent and added to the slide, incubated in a humidified box at 37℃ in the dark for 10 min, and the slide was washed 3 times after completion. The steps were the same as before. XTSA 520 dye was diluted 1:100 with signal amplification solution and added to the slide, incubated in a humidified box at room temperature in the dark for 10 min, and the slide was washed 3 times after completion. The steps were the same as before. CD80 was diluted 1:200 with antibody diluent and added to the slide, incubated in a humidified box in the dark for 1 hour, and the slide was washed 3 times after completion. The steps were the same as before. Dilute the AXB secondary antibody with antibody diluent at 1:3 and drop it on the slide. Incubate in a humidified chamber at 37°C in the dark for 10 min. Wash the slide 3 times after completion. The steps are the same as before. Dilute the XTSA 620 dye with signal amplification solution at 1:100 and drop it on the slide. Incubate in a humidified chamber at room temperature in the dark for 10 min. Wash the slide 3 times after completion. The steps are the same as before. DAPI is added to stain the cell nucleus and placed in the dark for 10 min. After completion, the slices are soaked in PBS for 5 min and washed 3 times. Add sealing agent to seal the slide and dry it. After sealing, the expression of each protein in the sample can be observed by the fluorescence scanning imaging system. The dilution ratio of CD206 is 1:500, and the rest of the steps are the same as before.

Dilute Ki-67 with antibody diluent at 1:500 and add it to the slide. Incubate in a humidified chamber 4 in the dark overnight. Wash the slide 3 times after completion. The steps are the same as before. Dilute the AXB secondary antibody with antibody diluent at 1:3 and add it to the slide. Incubate in a humidified box at 37°C in the dark for 10 min. Wash the slide 3 times after completion. The steps are the same as before. Dilute the XTSA 670 dye with signal amplification solution at 1:100 and add it to the slide. Incubate in a humidified box at room temperature in the dark for 10 min. Wash the slide 3 times after completion. The steps are the same as before. Add DAPI to stain the cell nucleus and place it in the dark for 10 min. After completion, soak the slice in PBS for 5 min and wash it 3 times. Add sealing agent to seal the slide and dry it. After sealing, the expression of each protein in the sample can be observed by the fluorescence scanning imaging system.

After fusion, images from five panels, each containing Ki-67, CD8, CD4, F4/80+CD80, and F4/80+CD206, were selected, and grayscale values (ImageJ_v1.8.0)​from three sections at different locations were statistically analyzed.

**Screening of effective active ingredients by HPLC-Q-TOF-MS/MS**

YFSJF-O, YFSJF-N and mouse drug-containing serum were subjected to triple quadrupole mass spectrometry component analysis to analyze the main active ingredients of NAP in YFSJF and explain the association mechanism between "ingredients and effects". The stock solution and NAP lyophilized powder were prepared, and the mouse drug-containing serum was prepared at the same time. The preparation method of drug-containing serum was as follows: the mice were fasted for 12 hours before administration, and the mice were given (gavage/tail vein) for 2 hours. About 250 μL of whole blood was collected from the retro-orbital venous plexus (eyeball removal) in a heparinized test tube, centrifuged at 4000 rpm for 10 min, and plasma was separated. 100 μL of each sample (300 μL in total) was added to a 1.5 mL centrifuge tube, vortexed for 1 min, mixed, and 3 times the volume of acetonitrile was added, vortexed for 1 min, and centrifuged at 4℃ (15000 rpm, 10 min). The supernatant was taken to a new 1.5 mL centrifuge tube, blown dry with high-purity N2 (or spin-dried at room temperature), and the remaining plasma was stored at -40℃. The detection equipment is Ultra-highperformance liquid chromatography system (UHPLC; LC40D X3 binary pump, SIL-40C autosampler, CTO-40C column oven; Shimadzu), triple quadrupole time-of-flight mass spectrometer (Q-TOF-MS/MS; ZenoTOF 7600 plus, AB Sciex). The mass spectrometry detection conditions are as follows:

**Table** The mass spectrometry detection conditions

| Time (min) | Flow Rate (mL/min) | %A | %B | Curve |
| --- | --- | --- | --- | --- |
| Initial | 0.300 | 95.0 | 5.0 | Initial |
| 12.00 | 0.300 | 47.0 | 53.0 | 6 |
| 32.00 | 0.300 | 5.0 | 95.0 | 6 |
| 35.00 | 0.300 | 5.0 | 95.0 | 6 |
| 37.00 | 0.300 | 95.0 | 5.0 | 6 |
| 40.00 | 0.300 | 95.0 | 5.0 | 6 |

Mass spectrometry condition parameters, A is 0.1% formic acid water, B is acetonitrile.

**Preparation of NAP**

Peimine, peiminine, peimisine, ginsenoside Re, ginsenoside Ro, rosmarinic acid, glycyrrhizic acid, ganoderic acid H and citric acid were first prepared into an anhydrous ethanol solution of 4.2 mg/mL. According to the results obtained by mass spectrometry, the proportions of different drugs were converted and prepared into different combinations of solutions of 4.2 mg/mL (for specific combinations, see Supplementary Materials Table S7). Then, the prepared solution was used to evaporate the alcohol by thin film dispersion method, and re-dissolved with an equal volume of deionized water to obtain NAP solution, which was stored at 4°C. The characterization method was the same as before.

**Molecular dynamics**

**Table** The number of molecules in simulation box.

| Type | Number |
| --- | --- |
| Peimine | 9 |
| Peiminine | 6 |
| Peimisine | 1 |
| Ginsenoside Re | 2 |
| Ginsenoside Ro | 8 |
| Rosmarinic acid | 1 |
| Glycyrrhizic acid | 9 |
| Ganoderic acid H | 7 |
| Citric acid | 1 |
| H_2_O | 22220 |

In the molecular dynamics simulation (MD simulation) stage, the Packmol tool was first used to construct a 10 nm × 10 nm × 10 nm cubic simulation box, and the molecules were randomly arranged according to the number of molecules in the above table. MD simulation was implemented using GROMACS 2020.6. The 3D structures of small molecules in the system were obtained from the Pubchem database, and the GAFF all-atom force field molecular topology file was constructed using Sobtop 1.0 (dev5) (http://sobereva.com/soft/Sobtop/). The water model used TIP3P. The simulation parameter settings included LINCS hydrogen bond constraints, 2 fs integration step, PME long-range electrostatic treatment, and 12 Å non-bonded cutoff radius (neighbor list updated every 10 steps). The temperature control (298.15 K) used the V-rescale coupler, and the pressure control (1 bar) applied the Parrinello-Rahman method. The simulation process includes: using the steepest descent method to minimize the energy of the system (emtol = 500) to eliminate close contact between atoms; performing NVT ensemble equilibrium for 100 ps at 298.15 K (temperature stability); performing NPT ensemble equilibrium for 100 ps at 1 bar pressure (pressure stability); after confirming the system equilibrium, the system is subjected to 50 ns of production simulation trajectory data collected every 10 ps, and the visualization analysis is completed collaboratively through the GROMACS built-in tools, PyMOL 2.6 and Discovery Studio Visualizer 2019.

**Molecular mass detection and calculation**

This supplementary material provides detailed calculations for determining the molecular quantities and mass percentages of nine monomeric constituents within a composite system with a total molecular weight of 1,292,000 g/mol. The calculation methodology is based on established mass ratios and molecular weights of individual components.

1. Total molecular mass of the composite system: 1,292,000 g/mol
2. Molecular weights and mass ratios of constituent monomers:

| Monomer | Molecular Weight (g/mol) | Mass Ratio |
| --- | --- | --- |
| Peimine | 431.7 | 9 |
| Peiminine | 429.6 | 6 |
| Peimisine | 427.6 | 1 |
| Ginsenoside Re | 947.2 | 2 |
| Ginsenoside Ro | 957.1 | 8 |
| Rosmarinic acid | 360.3 | 1 |
| Glycyrrhizic acid | 822.9 | 9 |
| Ganoderic acid H | 516.7 | 7 |
| Citric acid | 192.12 | 1 |

3. The molar ratios were calculated using the formula:

$$\text{n}_{\text{i}}\text{​=}\frac{\text{w}_{\text{i}}}{\text{M}_{\text{i}}}$$

Where *w*_i_ represents the mass ratio and M_i_ is the molecular weight of each monomer. The derived molar ratios were normalized to the smallest value (0.00211) and rounded to obtain practical integer ratios: 10 : 7 : 1 : 1 : 4 : 1 : 5 : 6 : 2.

4.The scaling factor $\text{k}$ was calculated using:

$$\text{k}\text{=​}\frac{\text{Total}\text{ }\text{Molecular}\text{ }\text{Weight}}{\text{∑}\text{(}\text{ni}\text{​×}\text{Mi}\text{​)}}\text{=}\frac{\text{20,486.84}}{\text{1,292,000}}\text{​}\text{=}\text{6}\text{3}\text{.06}$$

Molecular quantities were obtained by multiplying the integer ratios by the scaling factor and rounding to nearest integers.

5.Molecular Quantities and Mass Contributions

| Monomer | Quantity | Mass Contribution (g/mol) | Mass Percentage |
| --- | --- | --- | --- |
| Peimine | 631 | 272,372.70 | 21.10% |
| Peiminine | 441 | 189,453.60 | 14.67% |
| Peimisine | 63 | 26,938.80 | 2.09% |
| Ginsenoside Re | 63 | 59,673.60 | 4.62% |
| Ginsenoside Ro | 252 | 241,189.20 | 18.68% |
| Rosmarinic acid | 63 | 22,698.90 | 1.76% |
| Glycyrrhizic acid | 315 | 259,213.50 | 20.08% |
| Ganoderic acid H | 378 | 195,312.60 | 15.13% |
| Citric acid | 126 | 24,207.10 | 1.87% |
| Total | - | 1,291,060.00 | 100.00% |

6.The calculated total molecular mass (1,291,060.0 g/mol) shows excellent agreement with the experimental value (1,292,000 g/mol), with a minor deviation of 0.07%, confirming the reliability of our computational approach.

**Statistical analysis**

All data are expressed as mean ± standard deviation. Survival analysis was performed using the Log-rank (Mantel-Cox) test. Two groups were statistically analyzed using independent samples t-test and multiple groups were statistically analyzed using one-way ANOVA (Graphpad Pism9.5 software) followed by LSD and Duncan's multiple comparison test. For all cases, differences were considered statistically significant if p < 0.05 (*p < 0.05, **p < 0.01, ***p < 0.001 and ****p < 0.0001).

**Table S1.** Clinical patients’ information

| Medical number | Gender | Age | Date of diagnosis | Pathological diagnosis | TNM | Therapy | Last follow-up time | OS(Mouths) | Chinese medicine medication time (Months) | Death |
| --- | --- | --- | --- | --- | --- | --- | --- | --- | --- | --- |
| 611978 | Female | 63 | 2010-7 | Adenocarcinoma | T2aN2M1a ⅣA | Gefitinib | 2024-6 | 168 | 162 | N/A |
| 10271886 | Female | 70 | 2016-4-27 | Adenocarcinoma | T2bN2M0 ⅢA | Icotinib | 2024-10 | 102 | 102 | No |
| 10258322 | Male | 52 | 2012-4-25 | Adenocarcinoma | cT4NxM1 Ⅳ | Docetaxel + Nedaplatin | 2017-5 | 61 | 61 | Yes |
| 427612 | Male | 72 | 2016-1-2 | Lymphoepithelioma-like carcinoma | T4N3M0 Ⅲc | Docetaxel | 2018-10-20 | 33 | 33 | Yes |
| 496625 | Male | 70 | 2018-1-23 | Small cell carcinoma | T3N2M0 ⅢA | Etoposide + Cisplatin | 2022-4-4 | 51 | 51 | Yes |
| 13814101 | Male | 53 | 2021-12 | Small cell carcinoma | T1bN1M1 ⅣA | Etoposide + Cisplatin | 2014-7 | 31 | 17 | N/A |
| 12528582 | Male | 72 | 2020-1-19 | Small cell carcinoma and Squamous cell carcinoma | cT2bN2M0 ⅢA | Etoposide + Carboplatin | 2024-10-5 | 57 | 54 | No |
| 12591409 | Male | 58 | 2020-4 | Adenocarcinoma | T2aN3M1c2 ⅣB | Dabrafenib + Trametinib | 2024-9-9 | 55 | 45 | No |
| 13743339 | Male | 59 | 2021-11 | Small cell carcinoma | TxN3M1 ⅣA | Etoposide + Cisplatin + Durvalumab | 2024-8 | 33 | 24 | No |
| 12480615 | Male | 73 | 2019-12-23 | Squamous cell carcinoma | cT2aN1M1a ⅣA | Nab-Paclitaxel + Carboplatin | 2023-9-18 | 45 | 36 | No |
| 313618 | Male | 57 | 2011-12-27 | Adenocarcinoma | T3N2M1 Ⅳ | Paclitaxel liposomal + Cisplatin | 2018-9-11 | 93 | 92 | Yes |
| 10283142 | Male | 74 | 2019-8 | Squamous cell carcinoma | cT2N3M0 ⅢB | Nab-Paclitaxel + Cisplatin + Nivolumab | 2024-11 | 63 | 34 | No |
| 14654275 | Male | 63 | 2021-10-27 | Squamous cell carcinoma | cT4N3M0 ⅢC | Nab-Paclitaxel + Cisplatin + Sintilimab | 2024-11 | 37 | 31 | No |
| 6113177 | Male | 70 | 2020-9-11 | Adenocarcinoma | cT3N+M1 Ⅳ | Nab-Paclitaxel + Carboplatin + Pembrolizumab | 2024-10-11 | 49 | 45 | No |
| 14654275 | Male | 82 | 2020-12 | Squamous cell carcinoma | T3N0M1a ⅣA | Durvalumab + Apatinib | 2023-8-31 | 32 | 29 | N/A |
| 13534512 | Male | 57 | 2021-10-13 | Adenocarcinoma | T4NxM1a ⅣA | Nab-Paclitaxel + Cisplatin | 2024-11 | 37 | 33 | No |
| 13212272 | Female | 77 | 2021-4-26 | Adenocarcinoma | pTxNxM1c ⅣB | Icotinib | 2024-11-2 | 43 | 40 | No |
| 5975478 | Male | 65 | 2010-11 | Adenocarcinoma | rT0N3M0 ⅢB | No other treatments for postoperative recurrence | 2024-11 | 168 | 24 | No |
| 13696101 | Male | 67 | 2022-3 | Squamous cell carcinoma | T2aN2bM0 ⅢB | Docetaxel + Lobaplatin + Sintilimab | 2024-6-25 | 27 | 24 | No |
| 11974871 | Male | 69 | 2017-10-27 | Adenocarcinoma | rTxN2M1 ⅣA | Anlotinib | 2023-12 | 74 | 46 | Yes |
| 13566589 | Male | 66 | 2022-1-4 | Small cell carcinoma | cT4NxM1 Ⅳ | Etoposide + Carboplatin + Atezolizumab | 2024-9-26 | 35 | 33 | No |
| 2215310 | Female | 63 | 2011-11-9 | Adenocarcinoma | T4N3M1a Ⅳa | Gemcitabine + Nedaplatin + Endostar | 2018-7-17 | 80 | 80 | Yes |
| 10884128 | Male | 54 | 2016 | Adenocarcinoma | Ⅳ | Gefitinib | 2024-12-16 | 96 | 48 | No |
| 11204212 | Female | 36 | 2017-8 | Adenocarcinoma | T4N3M1c Ⅳ | Pemetrexed + Cisplatin | 2023-5 | 69 | 19 | N/A |
| 389177 | Male | 63 | 2014-9-30 | Small cell carcinoma | T4N2M0 ⅢA | Etoposide + Cisplatin + Endostar | 2017-9 | 36 | 36 | Yes |
| 12588527 | Male | 60 | 2017-1-10 | Squamous cell carcinoma | T2aN1M0 IIB | Docetaxel + Carboplatin + Endostar | 2024-7 | 90 | 46 | No |
| 11850074 | Male | 61 | 2018-7 | Adenocarcinoma | T4N3M1 ⅣB | Pemetrexed + carboplatin + bevacizumab | 2024-5-16 | 70 | 70 | No |
| 5575865 | Female | 59 | 2017-10-18 | Adenocarcinoma | cT4N3M1 Ⅳ | Pemetrexed + cisplatin | 2024-11-27 | 85 | 48 | No |
| 13696101 | Male | 67 | 2022-3 | Squamous cell carcinoma | T3N2bM0 ⅢB | Docetaxel + lobaplatin + Sintilimab | 2024-12-28 | 33 | 33 | No |
| 12510547 | Male | 71 | 2020-3-3 | Squamous cell carcinoma | cT3N0M1a ⅣA | Docetaxel + cisplatin | 2024-11-6 | 56 | 52 | No |
| 12607003 | Male | 74 | 2020-6-3 | Squamous cell carcinoma | T4N2M0 ⅢB | Paclitaxel + cisplatin | 2024-11 | 53 | 53 | No |


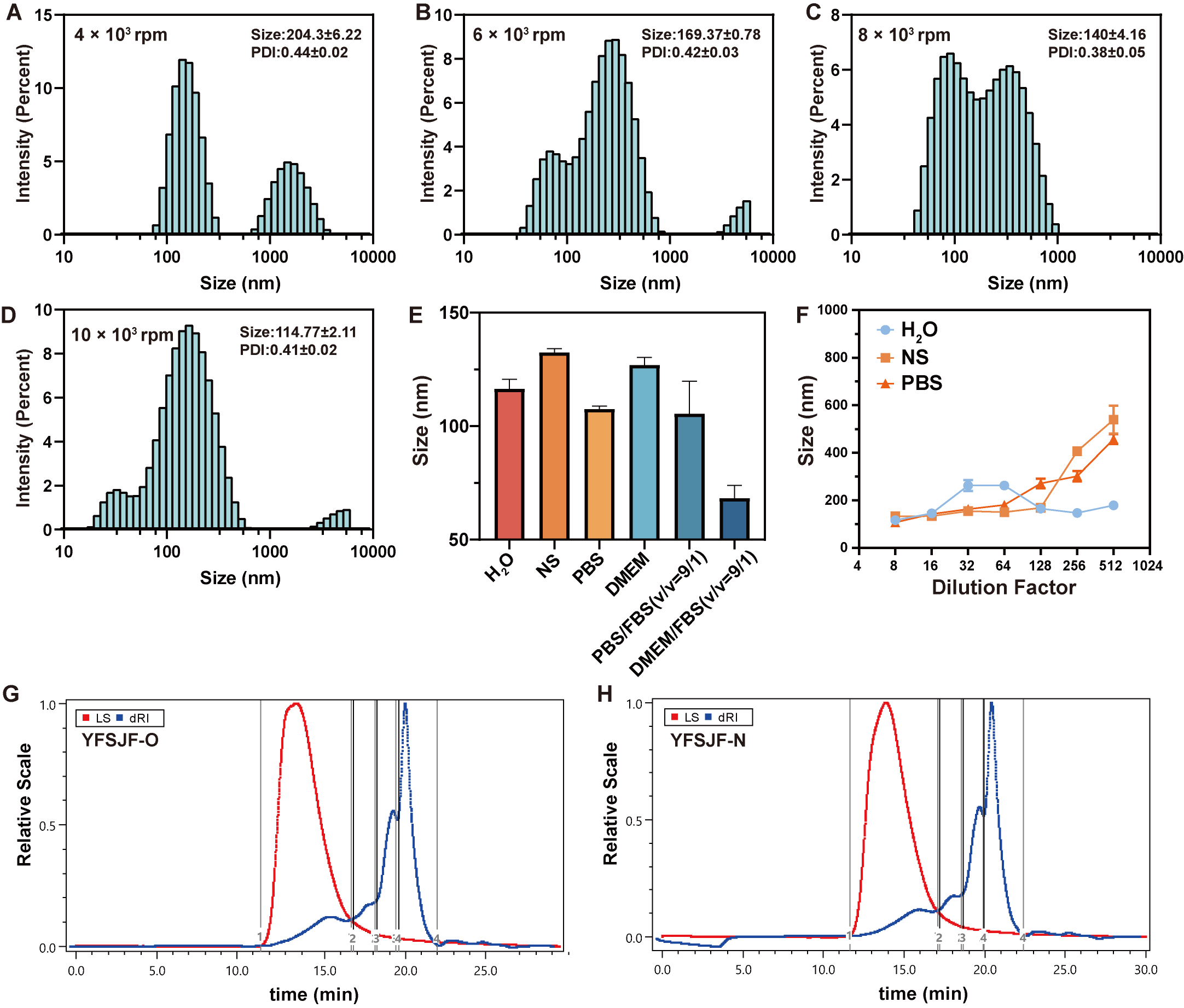


**Fig S1**. Characterization of YFSJF. (A)The particle size of the solution obtained by centrifugation at 4 × 10^3^ rpm for 30 min was 204.3 ± 6.22 nm, and the PDI was 0.44 ± 0.02. (B)The particle size of the solution obtained by centrifugation at 6 × 10^3^ rpm for 30 min was 169.37 ± 0.78 nm, and the PDI was 0.42 ± 0.03. (C)The particle size of the solution obtained by centrifugation at 8 × 10^3^ rpm for 30 min was 140 ± 4.16 nm, and the PDI was 0.38 ± 0.05. (D)The particle size of the solution obtained by centrifugation at 10 × 10^3^ rpm for 30 min was 114.77 ± 2.11 nm, and the PDI was 0.41 ± 0.02. (E)YFSJF-N medium stability testing. YFSJF-N was diluted to 1/8 of its original concentration using ultrapure water, NS, PBS, DMEM, PBS/FBS (v/v = 9/1), and DMEM/FBS (v/v = 9/1). The particle size was then measured. (F)YFSJF-N dilution stability test. YFSJF-N was diluted to 1/8, 1/16, 1/32, 1/64, 1/128, 1/256, and 1/512 of its original concentration using ultrapure water, NS, and PBS. The particle size was then measured. (G-H) YFSJF-O and YFSJF-N were detected using SEC-MALS.

**Fig S2**. Lewis cell survival rate at different concentrations after 72 hours of YFSJF-O and YFSJF-N administration.

**
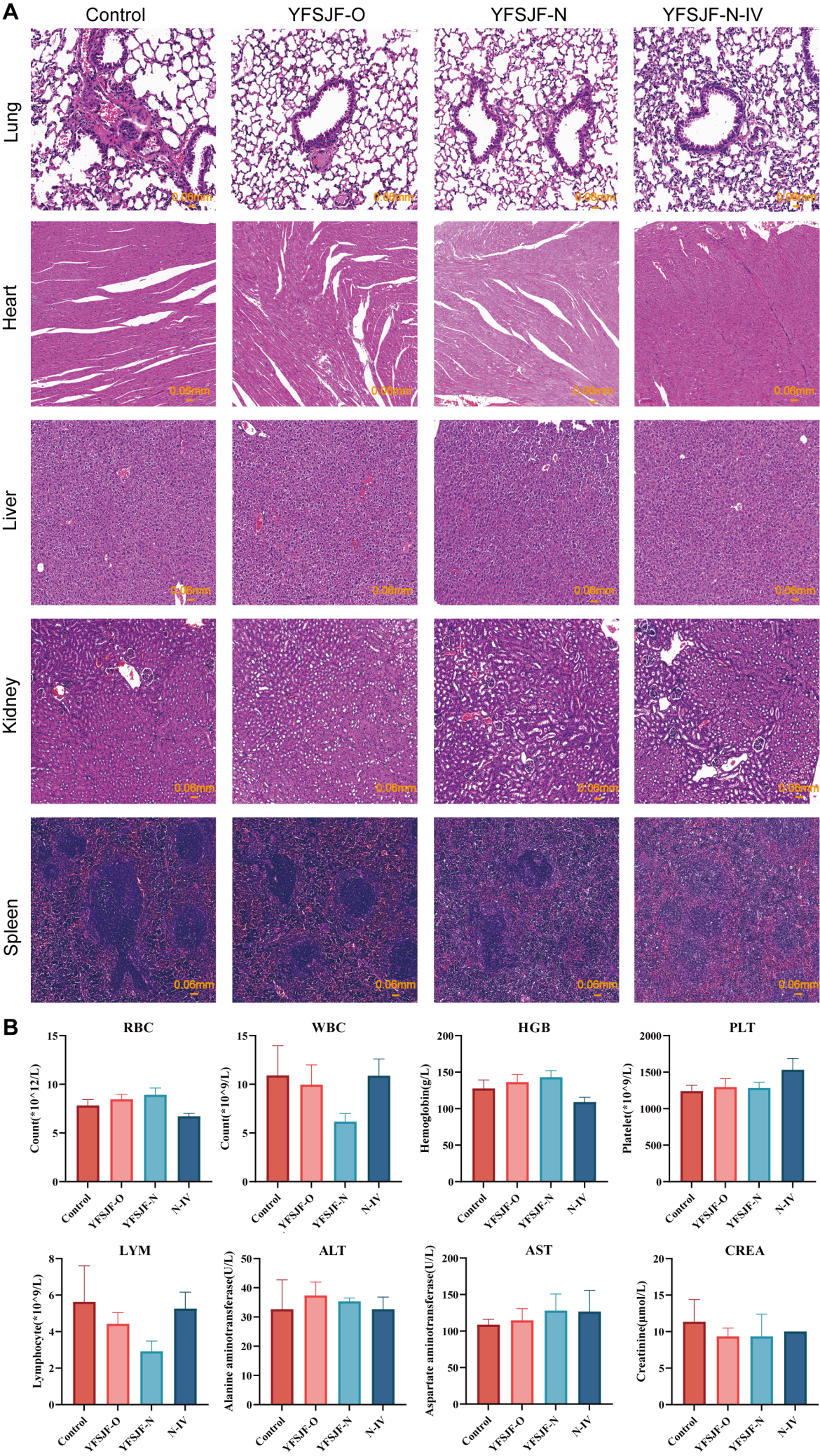
**

**Fig S3.** A show HE staining of lung, heart, liver, kidney and spleen of the four groups: Control, YFSJF-O, YFSJF-N and YFSJF-N-IV. B shows blood test values of red blood cells, white blood cells, hemoglobin, platelets, lymphocytes, ALT, AST and creatinine of the four groups.

**
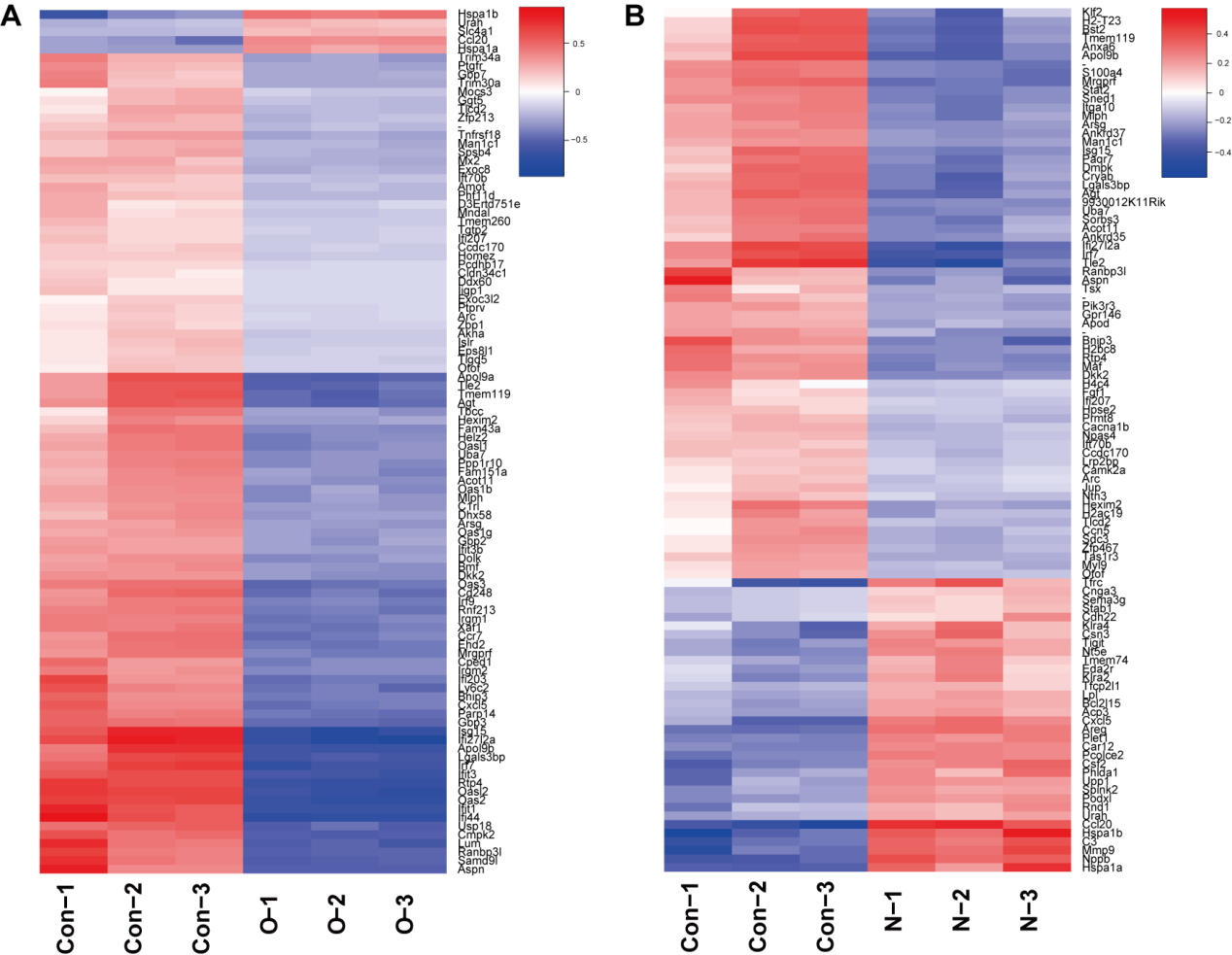
**

**Fig S4.** A is the differential gene expression between YFSJF-O and Control groups. B is the differential gene expression between YFSJF-N and Control.

**Table S2.** GO analysis data

| ID | description | name | ratio_in_study | ratio | up_in_study | down_in_study | ratio_in_pop | pvalue | fdr |
| --- | --- | --- | --- | --- | --- | --- | --- | --- | --- |
| GO:0110165 | cellular anatomical entity | CC | 565/615 | 0.9187 | 126 | 439 | 18615/21028 | 0.004573652 | 0.041284503 |
| GO:0009987 | cellular process | BP | 479/615 | 0.7789 | 113 | 366 | 15447/21028 | 0.007252807 | 0.050442679 |
| GO:0005488 | binding | MF | 458/615 | 0.7447 | 102 | 356 | 14045/21028 | 2.80E-05 | 0.000688568 |
| GO:0043226 | organelle | CC | 383/615 | 0.6228 | 88 | 295 | 12025/21028 | 0.00645545 | 0.048047803 |
| GO:0050789 | regulation of biological process | BP | 378/615 | 0.6146 | 86 | 292 | 12123/21028 | 0.032125191 | 0.139072575 |
| GO:0043229 | intracellular organelle | CC | 377/615 | 0.6130 | 88 | 289 | 11770/21028 | 0.004739328 | 0.042108057 |
| GO:0043227 | membrane-bounded organelle | CC | 347/615 | 0.5642 | 84 | 263 | 10819/21028 | 0.008319357 | 0.055802171 |
| GO:0005515 | protein binding | MF | 346/615 | 0.5626 | 84 | 262 | 9617/21028 | 1.59E-07 | 9.66E-06 |
| GO:0043231 | intracellular membrane-bounded organelle | CC | 322/615 | 0.5236 | 82 | 240 | 10001/21028 | 0.010566467 | 0.06616714 |
| GO:0005737 | cytoplasm | CC | 243/615 | 0.3951 | 50 | 193 | 7101/21028 | 0.001910752 | 0.022058141 |
| GO:0048518 | positive regulation of biological process | BP | 238/615 | 0.3870 | 60 | 178 | 6392/21028 | 9.56E-06 | 0.000300312 |
| GO:0019222 | regulation of metabolic process | BP | 228/615 | 0.3707 | 49 | 179 | 6738/21028 | 0.005190417 | 0.043821577 |
| GO:0048522 | positive regulation of cellular process | BP | 215/615 | 0.3496 | 56 | 159 | 5835/21028 | 7.10E-05 | 0.001525591 |
| GO:0050896 | response to stimulus | BP | 213/615 | 0.3463 | 57 | 156 | 5799/21028 | 9.56E-05 | 0.001908769 |
| GO:0048519 | negative regulation of biological process | BP | 208/615 | 0.3382 | 53 | 155 | 5568/21028 | 4.38E-05 | 0.001047667 |
| GO:0060255 | regulation of macromolecule metabolic process | BP | 204/615 | 0.3317 | 46 | 158 | 6239/21028 | 0.034806539 | 0.147666742 |
| GO:0031323 | regulation of cellular metabolic process | BP | 203/615 | 0.3301 | 44 | 159 | 5973/21028 | 0.007790388 | 0.05374101 |
| GO:0043167 | ion binding | MF | 195/615 | 0.3171 | 46 | 149 | 5694/21028 | 0.006846325 | 0.049022 |
| GO:0080090 | regulation of primary metabolic process | BP | 194/615 | 0.3154 | 42 | 152 | 5808/21028 | 0.018896895 | 0.096882271 |
| GO:0048523 | negative regulation of cellular process | BP | 188/615 | 0.3057 | 48 | 140 | 5049/21028 | 0.000153488 | 0.002800745 |
| GO:0003824 | catalytic activity | MF | 187/615 | 0.3041 | 41 | 146 | 5658/21028 | 0.031095794 | 0.136708709 |
| GO:0051171 | regulation of nitrogen compound metabolic process | BP | 186/615 | 0.3024 | 43 | 143 | 5626/21028 | 0.03126873 | 0.137113784 |
| GO:0032502 | developmental process | BP | 183/615 | 0.2976 | 47 | 136 | 5427/21028 | 0.01651468 | 0.089252906 |
| GO:0016043 | cellular component organization | BP | 172/615 | 0.2797 | 42 | 130 | 5070/21028 | 0.016713048 | 0.090038233 |
| GO:0071840 | cellular component organization or biogenesis | BP | 172/615 | 0.2797 | 42 | 130 | 5189/21028 | 0.035926224 | 0.149063087 |
| GO:0048583 | regulation of response to stimulus | BP | 170/615 | 0.2764 | 40 | 130 | 4178/21028 | 3.08E-06 | 0.000113786 |
| GO:0010468 | regulation of gene expression | BP | 165/615 | 0.2683 | 34 | 131 | 4700/21028 | 0.00570325 | 0.046755625 |
| GO:0006950 | response to stress | BP | 153/615 | 0.2488 | 42 | 111 | 3032/21028 | 1.91E-11 | 5.40E-09 |
| GO:0010646 | regulation of cell communication | BP | 150/615 | 0.2439 | 32 | 118 | 3570/21028 | 2.73E-06 | 0.000102948 |
| GO:0023051 | regulation of signaling | BP | 150/615 | 0.2439 | 32 | 118 | 3588/21028 | 3.59E-06 | 0.000127088 |

**
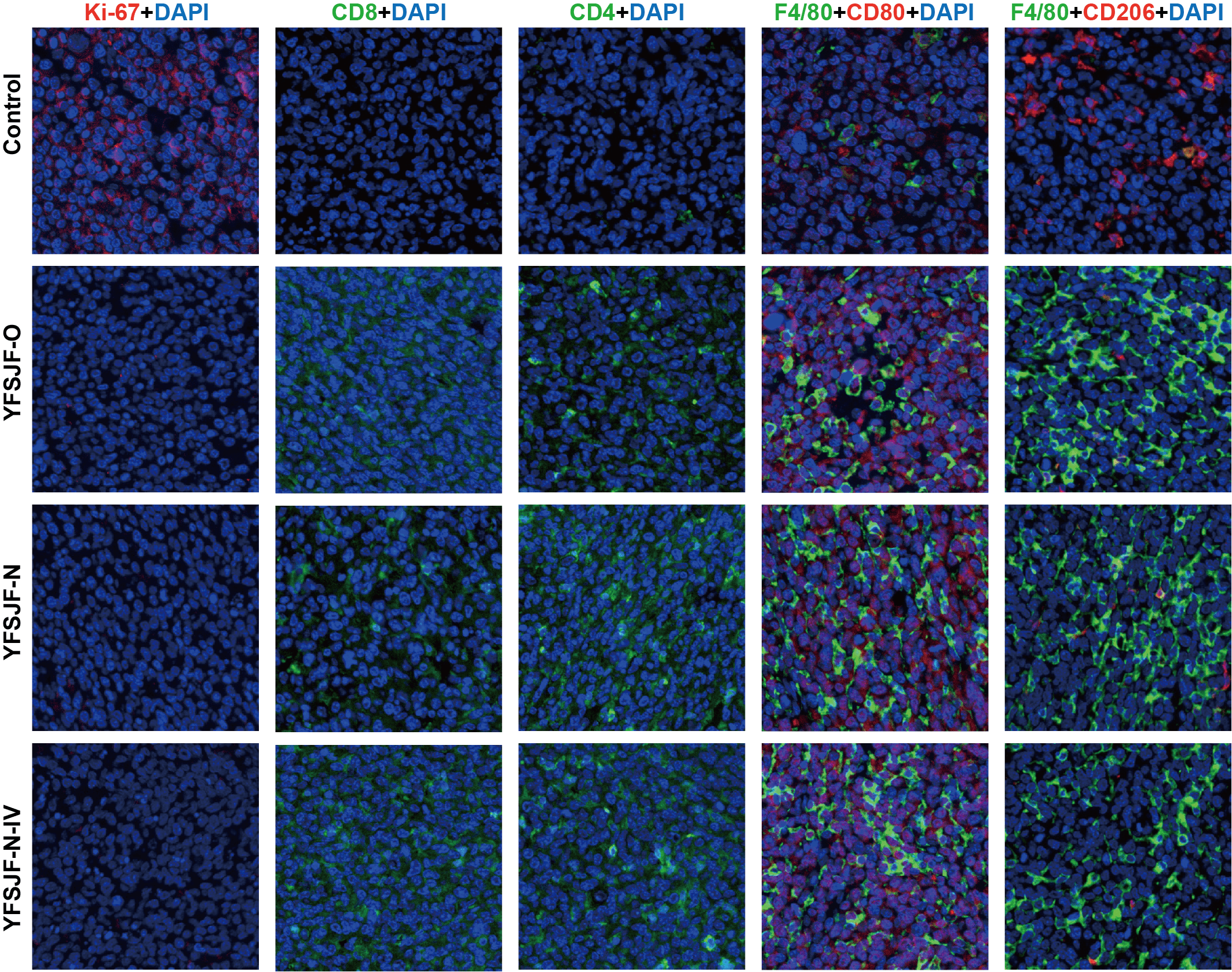
Fig S5.** Immunofluorescence staining of tumor tissues. Control, YFSJF-O, YFSJF-N, and YFSJF-N-IV tumor tissues from the animal experiments were immunostained for Ki-67, CD8, CD4, F4/80+CD80, and F4/80+CD206 expression, respectively.

**Table S3.** Mass spectrometry response value data

| Chinese medicine name | Monomer name | Chemical formula | Intensity | | | | |
| --- | --- | --- | --- | --- | --- | --- | --- |
|  |  |  | YFSJF-O | YFSJF-N | YFSJF-O-**Oral gavage** | YFSJF-N-**Oral gavage** | YFSJF-N-IV |
| Fritillaria thunbergii Miq. | Peimine | C27H45NO3 | 301535 | 143567 | 93395 | 86135 | 113614 |
|  | Peiminine | C27H43NO3 | 167701 | 100305 | 122941 | 130425 | 99425 |
|  | Peimisine | C27H41NO3 | 33735 | 16769 | 2216 | 2088 | 3725 |
| Panax quinquefolium L. | Ginsenoside Re | C48H82O18 | 86387 | 31905 | n.a | n.a | 30115 |
|  | Ginsenoside Rg1 | C42H72O14 | n.a | n.a | n.a | n.a | n.a |
|  | Pseudoginsenoside F11 | C42H72O14 | 9645 | 4591 | n.a | n.a | 182 |
|  | Ginsenoside Rc | C53H90O22 | 9659 | 22517 | n.a | n.a | n.a |
|  | Ginsenoside Ro | C48H76O19 | 186873 | 139685 | 2881 | 940 | 202622 |
|  | Ginsenoside Rb1 | C54H92O23 | 258002 | 152125 | n.a | n.a | n.a |
| Bombyx batryticatus | Beauvericin | C45H57N3O9 | n.a | n.a | n.a | n.a | n.a |
|  | β-sitosterol | C29H50O | n.a | n.a | n.a | n.a | n.a |
|  | Stigmasterol | C29H48O | n.a | n.a | n.a | n.a | n.a |
|  | Citric acid | C6H8O7 | 211312 | 20228 | 40505 | 46337 | 89264 |
| Sarcandra glabra (Thunb.) Nakai | Astilbin | C21H22O11 | 276 | 0 | n.a | n.a | n.a |
|  | Rosmarinic acid | C18H16O8 | 86148 | 17188 | 2705 | n.a | n.a |
|  | Isofraxin | C11H10O5 | 366095 | 17420 | n.a | n.a | n.a |
|  | Chlorogenic acid | C16H18O9 | 13879 | 3042 | n.a | n.a | n.a |
| Ranunculus ternatus Thunb. | Chrysin | C15H10O4 | 172 | 150 | n.a | n.a | n.a |
|  | Rutin | C27H30O16 | 1855 | 511 | n.a | n.a | n.a |
| Pinellia ternata (Thunb.) Breit. | Lysophosphatidylcholine | C24H50NO7P | 1603 | n.a | 8200539 | 8164787 | 8539004 |
|  | Trigonelline | C7H8ClNO2 | n.a | n.a | n.a | n.a | n.a |
|  | Glycyrrhizin | C15H12O4 | 10880 | 1985 | n.a | n.a | n.a |
|  | Glycyrrhizic acid | C42H62O16 | 88405 | 152686 | n.a | n.a | 20016 |
|  | Glycyrrhetinic acid | C30H46O4 | 12449 | 15465 | n.a | n.a | n.a |
| Cremastra appendiculata (D.Don) Makino | Bletilla striata glycoside | C34H46O17 | n.a | n.a | n.a | n.a | n.a |
|  | Colchicine | C22H25NO6 | n.a | n.a | n.a | n.a | n.a |
|  | Pinusinol-pyranoglucoside | C26H32O11 | n.a | n.a | n.a | n.a | n.a |
|  | Luteolin | C21H18O12 | 12342 | 2580 | n.a | n.a | n.a |
| Ganoderma lucidum (Leyss.ex Fr.) Karst. | Ganoderic acid A | C30H44O7 | 371094 | 162367 | n.a | n.a | n.a |
|  | Ganoderic acid H | C32H44O9 | 312191 | 124735 | 157468 | 278612 | 127721 |
|  | Ganoderic acid C2 | C30H46O7 | 13553 | 60159 | n.a | n.a | n.a |

The “n.a.” is “not applicable”.

**Table S4.** Energy: the name of the energy term; Average: the average value of the energy; Err.Est.: Error Estimate, indicating the uncertainty of the average value; RMSD: Root Mean Square Deviation, indicating the fluctuation of the energy term; Tot-Drift: Total Drift, indicating the cumulative change of energy during the simulation; all parameters are in kJ/mol.

| **Energy** | **Average** | **Err.Est.** | **RMSD** | **Tot-Drift** |
| --- | --- | --- | --- | --- |
| Coulomb (SR) | -8464.65 | 46 | 128.111 | -289.262 |
| LJ (SR) | -6156.19 | 260 | 621.146 | -1528.86 |

**Table S5.** Nanoprescription

| Combination number | Monomer name | | | | | | | | | |
| --- | --- | --- | --- | --- | --- | --- | --- | --- | --- | --- |
|  | Peimine | | Peiminine | Peimisine | GinsenosideRe | GinsenosideRo | Rosmarinic acid | Glycyrrhizic acid | Ganoderic acid H | Citric acid |
|  | 9 | 6 | | 1 | 2 | 8 | 1 | 9 | 7 | 1 |
| N-1 | √ | √ | | √ | √ | √ | √ | √ | √ | √ |
| N-2 | × | √ | | √ | √ | √ | √ | √ | √ | √ |
| N-3 | √ | × | | √ | √ | √ | √ | √ | √ | √ |
| N-4 | √ | √ | | × | √ | √ | √ | √ | √ | √ |
| N-5 | √ | √ | | √ | × | √ | √ | √ | √ | √ |
| N-6 | √ | √ | | √ | √ | × | √ | √ | √ | √ |
| N-7 | √ | √ | | √ | √ | √ | × | √ | √ | √ |
| N-8 | √ | √ | | √ | √ | √ | √ | × | √ | √ |
| N-9 | √ | √ | | √ | √ | √ | √ | √ | × | √ |
| N-10 | √ | √ | | √ | √ | √ | √ | √ | √ | × |
| N-11 | × | × | | √ | √ | √ | √ | √ | √ | √ |
| N-12 | × | √ | | × | √ | √ | √ | √ | √ | √ |
| N-13 | × | √ | | √ | × | √ | √ | √ | √ | √ |
| N-14 | × | √ | | √ | √ | √ | × | √ | √ | √ |
| N-15 | × | √ | | √ | √ | √ | √ | × | √ | √ |
| N-16 | × | √ | | √ | √ | √ | √ | √ | × | √ |
| N-17 | × | √ | | √ | √ | √ | √ | √ | √ | × |
| N-18 | √ | × | | × | √ | √ | √ | √ | √ | √ |
| N-19 | √ | × | | √ | × | √ | √ | √ | √ | √ |
| N-20 | √ | × | | √ | √ | √ | × | √ | √ | √ |
| N-21 | √ | × | | √ | √ | √ | √ | × | √ | √ |
| N-22 | √ | × | | √ | √ | √ | √ | √ | × | √ |
| N-23 | √ | × | | √ | √ | √ | √ | √ | √ | × |
| N-24 | √ | √ | | × | × | √ | √ | √ | √ | √ |
| N-25 | √ | √ | | × | √ | √ | × | √ | √ | √ |
| N-26 | √ | √ | | × | √ | √ | √ | × | √ | √ |
| N-27 | √ | √ | | × | √ | √ | √ | √ | × | √ |
| N-28 | √ | √ | | × | √ | √ | √ | √ | √ | × |
| N-29 | √ | √ | | √ | × | √ | × | √ | √ | √ |
| N-30 | √ | √ | | √ | × | √ | √ | × | √ | √ |
| N-31 | √ | √ | | √ | × | √ | √ | √ | × | √ |
| N-32 | √ | √ | | √ | × | √ | √ | √ | √ | × |
| N-33 | √ | √ | | √ | √ | √ | × | × | √ | √ |
| N-34 | √ | √ | | √ | √ | √ | × | √ | × | √ |
| N-35 | √ | √ | | √ | √ | √ | × | √ | √ | × |
| N-36 | √ | √ | | √ | √ | √ | √ | × | × | √ |
| N-37 | √ | √ | | √ | √ | √ | √ | × | √ | × |
| N-38 | √ | √ | | √ | √ | √ | √ | √ | × | × |
| N-39 | × | × | | × | √ | √ | √ | √ | √ | √ |
| N-40 | × | √ | | × | × | √ | √ | √ | √ | √ |
| N-41 | × | √ | | √ | × | √ | × | √ | √ | √ |
| N-42 | × | √ | | √ | √ | √ | × | × | √ | √ |
| N-43 | × | √ | | √ | √ | √ | √ | × | × | √ |
| N-44 | × | √ | | √ | √ | √ | √ | √ | × | × |
| N-45 | × | × | | √ | × | √ | √ | √ | √ | √ |
| N-46 | × | √ | | × | √ | √ | × | √ | √ | √ |
| N-47 | × | √ | | √ | × | √ | √ | × | √ | √ |
| N-48 | × | √ | | √ | √ | √ | × | √ | × | √ |
| N-49 | × | √ | | √ | √ | √ | √ | × | √ | × |
| N-50 | × | × | | √ | √ | √ | × | √ | √ | √ |
| N-51 | × | √ | | × | √ | √ | √ | × | √ | √ |
| N-52 | × | √ | | √ | × | √ | √ | √ | × | √ |
| N-53 | × | √ | | √ | √ | √ | × | √ | √ | × |
| N-54 | × | × | | √ | √ | √ | √ | × | √ | √ |
| N-55 | × | √ | | × | √ | √ | √ | √ | × | √ |
| N-56 | × | √ | | √ | × | √ | √ | √ | √ | × |
| N-57 | × | × | | √ | √ | √ | √ | √ | × | √ |
| N-58 | × | √ | | × | √ | √ | √ | √ | √ | × |
| N-59 | × | × | | √ | √ | √ | √ | √ | √ | × |
| N-60 | √ | × | | × | × | √ | √ | √ | √ | √ |
| N-61 | √ | × | | √ | × | √ | × | √ | √ | √ |
| N-62 | √ | × | | √ | √ | √ | × | × | √ | √ |
| N-63 | √ | × | | √ | √ | √ | √ | × | × | √ |
| N-64 | √ | × | | √ | √ | √ | √ | √ | × | × |
| N-65 | √ | × | | × | √ | √ | × | √ | √ | √ |
| N-66 | √ | × | | √ | × | √ | √ | × | √ | √ |
| N-67 | √ | × | | √ | √ | √ | × | √ | × | √ |
| N-68 | √ | × | | √ | √ | √ | √ | × | √ | × |
| N-69 | √ | × | | × | √ | √ | √ | × | √ | √ |
| N-70 | √ | × | | √ | × | √ | √ | √ | × | √ |
| N-71 | √ | × | | √ | √ | √ | × | √ | √ | × |
| N-72 | √ | × | | × | √ | √ | √ | √ | × | √ |
| N-73 | √ | × | | √ | × | √ | √ | √ | √ | × |
| N-74 | √ | × | | × | √ | √ | √ | √ | √ | × |
| N-75 | √ | √ | | × | × | √ | × | √ | √ | √ |
| N-76 | √ | √ | | × | √ | √ | × | × | √ | √ |
| N-77 | √ | √ | | × | √ | √ | √ | × | × | √ |
| N-78 | √ | √ | | × | √ | √ | √ | √ | × | × |
| N-79 | √ | √ | | × | × | √ | √ | × | √ | √ |
| N-80 | √ | √ | | × | √ | √ | × | √ | × | √ |
| N-81 | √ | √ | | × | √ | √ | √ | × | √ | × |
| N-82 | √ | √ | | × | × | √ | √ | √ | × | √ |
| N-83 | √ | √ | | × | √ | √ | × | √ | √ | × |
| N-84 | √ | √ | | × | × | √ | √ | √ | √ | × |
| N-85 | √ | √ | | √ | × | √ | × | × | √ | √ |
| N-86 | √ | √ | | √ | × | √ | √ | × | × | √ |
| N-87 | √ | √ | | √ | × | √ | √ | √ | × | × |
| N-88 | √ | √ | | √ | × | √ | × | √ | × | √ |
| N-89 | √ | √ | | √ | × | √ | √ | × | √ | × |
| N-90 | √ | √ | | √ | × | √ | × | √ | √ | × |
| N-91 | √ | √ | | √ | √ | √ | × | × | × | √ |
| N-92 | √ | √ | | √ | √ | √ | × | √ | × | × |
| N-93 | √ | √ | | √ | √ | √ | × | × | √ | × |
| N-94 | √ | √ | | √ | √ | √ | √ | × | × | × |
| N-95 | × | × | | × | × | √ | √ | √ | √ | √ |
| N-96 | × | √ | | × | × | √ | × | √ | √ | √ |
| N-97 | × | √ | | √ | × | √ | × | × | √ | √ |
| N-98 | × | √ | | √ | √ | √ | × | × | × | √ |
| N-99 | × | √ | | √ | √ | √ | √ | × | × | × |
| N-100 | × | × | | √ | × | √ | × | √ | √ | √ |
| N-101 | × | × | | √ | √ | √ | × | × | √ | √ |
| N-102 | × | × | | √ | √ | √ | √ | × | × | √ |
| N-103 | × | × | | √ | √ | √ | √ | √ | × | × |
| N-104 | × | × | | √ | × | √ | √ | × | √ | √ |
| N-105 | × | × | | √ | × | √ | √ | √ | × | √ |
| N-106 | × | × | | √ | × | √ | √ | √ | √ | × |
| N-107 | × | × | | √ | √ | √ | × | √ | × | √ |
| N-108 | × | × | | √ | √ | √ | × | √ | √ | × |
| N-109 | × | × | | √ | √ | √ | √ | × | √ | × |
| N-110 | × | × | | × | √ | √ | × | √ | √ | √ |
| N-111 | × | × | | × | √ | √ | √ | × | √ | √ |
| N-112 | × | × | | × | √ | √ | √ | √ | × | √ |
| N-113 | × | × | | × | √ | √ | √ | √ | √ | × |
| N-114 | × | √ | | × | √ | √ | × | × | √ | √ |
| N-115 | × | √ | | × | √ | √ | √ | × | × | √ |
| N-116 | × | √ | | × | √ | √ | √ | √ | × | × |
| N-117 | × | √ | | × | √ | √ | × | √ | × | √ |
| N-118 | × | √ | | × | √ | √ | √ | × | √ | × |
| N-119 | × | √ | | × | √ | √ | × | √ | √ | × |
| N-120 | × | √ | | × | × | √ | √ | × | √ | √ |
| N-121 | × | √ | | × | × | √ | √ | √ | × | √ |
| N-122 | × | √ | | × | × | √ | √ | √ | √ | × |
| N-123 | × | √ | | √ | × | √ | √ | × | × | √ |
| N-124 | × | √ | | √ | × | √ | √ | √ | × | × |
| N-125 | × | √ | | √ | × | √ | √ | × | √ | × |
| N-126 | × | √ | | √ | × | √ | × | √ | × | √ |
| N-127 | × | √ | | √ | × | √ | × | √ | √ | × |
| N-128 | × | √ | | √ | √ | √ | × | √ | × | × |
| N-129 | × | √ | | √ | √ | √ | × | × | √ | × |
| N-130 | √ | × | | × | × | √ | × | √ | √ | √ |
| N-131 | √ | × | | √ | × | √ | × | × | √ | √ |
| N-132 | √ | × | | √ | √ | √ | × | × | × | √ |
| N-133 | √ | × | | √ | √ | √ | √ | × | × | × |
| N-134 | √ | × | | × | √ | √ | × | × | √ | √ |
| N-135 | √ | × | | × | √ | √ | √ | × | × | √ |
| N-136 | √ | × | | × | √ | √ | √ | √ | × | × |
| N-137 | √ | × | | × | × | √ | √ | × | √ | √ |
| N-138 | √ | × | | × | × | √ | √ | √ | × | √ |
| N-139 | √ | × | | × | × | √ | √ | √ | √ | × |
| N-140 | √ | × | | × | √ | √ | × | √ | × | √ |
| N-141 | √ | × | | × | √ | √ | √ | × | √ | × |
| N-142 | √ | × | | × | √ | √ | × | √ | √ | × |
| N-143 | √ | × | | √ | × | √ | √ | × | × | √ |
| N-144 | √ | × | | √ | × | √ | √ | √ | × | × |
| N-145 | √ | × | | √ | × | √ | × | √ | × | √ |
| N-146 | √ | × | | √ | × | √ | √ | × | √ | × |
| N-147 | √ | × | | √ | × | √ | × | √ | √ | × |
| N-148 | √ | × | | √ | √ | √ | × | × | √ | × |
| N-149 | √ | × | | √ | √ | √ | × | √ | × | × |
| N-150 | √ | √ | | × | × | √ | × | × | √ | √ |
| N-151 | √ | √ | | × | √ | √ | × | × | × | √ |
| N-152 | √ | √ | | × | √ | √ | √ | × | × | × |
| N-153 | √ | √ | | × | × | √ | √ | × | × | √ |
| N-154 | √ | √ | | × | × | √ | √ | √ | × | × |
| N-155 | √ | √ | | × | × | √ | × | √ | × | √ |
| N-156 | √ | √ | | × | × | √ | √ | × | √ | × |
| N-157 | √ | √ | | × | × | √ | × | √ | √ | × |
| N-158 | √ | √ | | × | √ | √ | × | × | √ | × |
| N-159 | √ | √ | | × | √ | √ | × | √ | × | × |
| N-160 | √ | √ | | √ | × | √ | × | × | × | √ |
| N-161 | √ | √ | | √ | × | √ | √ | × | × | × |
| N-162 | √ | √ | | √ | × | √ | × | √ | × | × |
| N-163 | √ | √ | | √ | × | √ | × | × | √ | × |
| N-164 | √ | √ | | √ | √ | √ | × | × | × | × |
| N-165 | × | × | | × | × | √ | × | √ | √ | √ |
| N-166 | × | × | | √ | × | √ | × | × | √ | √ |
| N-167 | × | × | | √ | √ | √ | × | × | × | √ |
| N-168 | × | × | | √ | √ | √ | √ | × | × | × |
| N-169 | × | × | | × | √ | √ | × | × | √ | √ |
| N-170 | × | × | | × | √ | √ | √ | × | × | √ |
| N-171 | × | × | | × | √ | √ | √ | √ | × | × |
| N-172 | × | × | | × | × | √ | √ | × | √ | √ |
| N-173 | × | × | | × | × | √ | √ | √ | × | √ |
| N-174 | × | × | | × | × | √ | √ | √ | √ | × |
| N-175 | × | × | | × | √ | √ | × | √ | × | √ |
| N-176 | × | × | | × | √ | √ | √ | × | √ | × |
| N-177 | × | × | | √ | × | √ | × | √ | × | √ |
| N-178 | × | × | | √ | √ | √ | × | × | √ | × |
| N-179 | × | × | | √ | × | √ | × | √ | √ | × |
| N-180 | × | × | | √ | × | √ | √ | × | √ | × |
| N-181 | × | × | | × | √ | √ | × | √ | √ | × |
| N-182 | × | × | | √ | √ | √ | × | √ | × | × |
| N-183 | × | × | | √ | × | √ | √ | √ | × | × |
| N-184 | × | × | | √ | × | √ | √ | × | × | √ |
| N-185 | × | √ | | √ | × | √ | × | × | × | √ |
| N-186 | × | √ | | √ | × | √ | × | × | √ | × |
| N-187 | × | √ | | √ | × | √ | × | √ | × | × |
| N-188 | × | √ | | √ | × | √ | √ | × | × | × |
| N-189 | × | √ | | √ | √ | √ | × | × | × | × |
| N-190 | × | √ | | × | √ | √ | × | × | × | √ |
| N-191 | × | √ | | × | √ | √ | × | × | √ | × |
| N-192 | × | √ | | × | √ | √ | × | √ | × | × |
| N-193 | × | √ | | × | √ | √ | √ | × | × | × |
| N-194 | × | √ | | × | × | √ | √ | × | × | √ |
| N-195 | × | √ | | × | × | √ | √ | × | √ | × |
| N-196 | × | √ | | × | × | √ | √ | √ | × | × |
| N-197 | × | √ | | × | × | √ | × | √ | × | √ |
| N-198 | × | √ | | × | × | √ | × | √ | √ | × |
| N-199 | × | √ | | × | × | √ | × | × | √ | √ |
| N-200 | √ | × | | × | × | √ | × | × | √ | √ |
| N-201 | √ | × | | √ | × | √ | × | × | × | √ |
| N-202 | √ | × | | √ | √ | √ | × | × | × | × |
| N-203 | √ | × | | × | √ | √ | × | × | × | √ |
| N-204 | √ | × | | × | √ | √ | √ | × | × | × |
| N-205 | √ | × | | × | × | √ | √ | × | × | √ |
| N-206 | √ | × | | × | × | √ | √ | √ | × | × |
| N-207 | √ | × | | × | × | √ | × | √ | × | √ |
| N-208 | √ | × | | × | × | √ | √ | × | √ | × |
| N-209 | √ | × | | × | × | √ | × | √ | √ | × |
| N-210 | √ | × | | √ | × | √ | × | √ | × | × |
| N-211 | √ | × | | √ | × | √ | √ | × | × | × |
| N-212 | √ | × | | √ | × | √ | × | × | √ | × |
| N-213 | √ | × | | × | √ | √ | × | × | √ | × |
| N-214 | √ | × | | × | √ | √ | × | √ | × | × |
| N-215 | √ | √ | | √ | × | √ | × | × | × | × |
| N-216 | √ | √ | | × | √ | √ | × | × | × | × |
| N-217 | √ | √ | | × | × | √ | √ | × | × | × |
| N-218 | √ | √ | | × | × | √ | × | √ | × | × |
| N-219 | √ | √ | | × | × | √ | × | × | √ | × |
| N-220 | √ | √ | | × | × | √ | × | × | × | √ |
| N-221 | × | √ | | × | × | √ | × | × | × | √ |
| N-222 | × | √ | | × | × | √ | × | × | √ | × |
| N-223 | × | √ | | × | × | √ | × | √ | × | × |
| N-224 | × | √ | | × | × | √ | √ | × | × | × |
| N-225 | × | √ | | × | √ | √ | × | × | × | × |
| N-226 | × | √ | | √ | × | √ | × | × | × | × |
| N-227 | × | × | | √ | × | √ | × | × | × | √ |
| N-228 | × | × | | √ | × | √ | × | × | √ | × |
| N-229 | × | × | | √ | × | √ | × | √ | × | × |
| N-230 | × | × | | √ | × | √ | √ | × | × | × |
| N-231 | × | × | | √ | √ | √ | × | × | × | × |
| N-232 | × | × | | × | √ | √ | × | × | × | √ |
| N-233 | × | × | | × | √ | √ | × | × | √ | × |
| N-234 | × | × | | × | √ | √ | × | √ | × | × |
| N-235 | × | × | | × | √ | √ | √ | × | × | × |
| N-236 | × | × | | × | × | √ | √ | × | × | √ |
| N-237 | × | × | | × | × | √ | √ | × | √ | × |
| N-238 | × | × | | × | × | √ | √ | √ | × | × |
| N-239 | × | × | | × | × | √ | × | √ | × | √ |
| N-240 | × | × | | × | × | √ | × | √ | √ | × |
| N-241 | × | × | | × | × | √ | × | × | √ | √ |
| N-242 | √ | × | | √ | × | √ | × | × | × | × |
| N-243 | √ | × | | × | √ | √ | × | × | × | × |
| N-244 | √ | × | | × | × | √ | √ | × | × | × |
| N-245 | √ | × | | × | × | √ | × | √ | × | × |
| N-246 | √ | × | | × | × | √ | × | × | √ | × |
| N-247 | √ | × | | × | × | √ | × | × | × | √ |
| N-248 | √ | √ | | × | × | √ | × | × | × | × |

**Table S6.** Summary of monomer ratios.

| Monomer | Quantity | Mass Contribution (g/mol) | Mass Percentage |
| --- | --- | --- | --- |
| Peimine | 631 | 272,372.70 | 21.10% |
| Peiminine | 441 | 189,453.60 | 14.67% |
| Peimisine | 63 | 26,938.80 | 2.09% |
| Ginsenoside Re | 63 | 59,673.60 | 4.62% |
| Ginsenoside Ro | 252 | 241,189.20 | 18.68% |
| Rosmarinic acid | 63 | 22,698.90 | 1.76% |
| Glycyrrhizic acid | 315 | 259,213.50 | 20.08% |
| Ganoderic acid H | 378 | 195,312.60 | 15.13% |
| Citric acid | 126 | 24,207.10 | 1.87% |
| Total | - | 1,291,060.00 | 100.00% |

**Table S7.** NAP characterization results

| Sample | Zeta Size（d.nm） | PDI | Zeta Potential（mV） |
| --- | --- | --- | --- |
| N-1 | 126.83±1.21 | 0.24±0.01 | -2.3±1.38 |
| N-2 | 120.93±10.69 | 0.79±0.16 | -8.07±3.14 |
| N-3 | 86.44±15.78 | 0.85±0.13 | -0.98±0.35 |
| N-4 | 242.43±31.06 | 0.14±0.07 | -7.37±0.61 |
| N-5 | 127.73±21.25 | 0.52±0.08 | -6.95±0.6 |
| N-6 | 156.97±10.87 | 0.37±0.08 | -12±0.53 |
| N-7 | 173.73±33.19 | 0.23±0.06 | -17.33±1.51 |
| N-8 | 155.83±5.52 | 0.35±0.01 | -30.2±3.44 |
| N-9 | 112.27±18.31 | 0.39±0.07 | -16.33±3.19 |
| N-10 | 161.83±15.32 | 0.43±0.04 | -9.16±1.66 |
| N-11 | 109.4±19.29 | 0.45±0.02 | -5.02±1.94 |
| N-12 | 136.27±11.58 | 0.36±0.07 | -9.15±2.99 |
| N-13 | 117.63±7.54 | 0.48±0.03 | -2.96±0.68 |
| N-14 | 158.03±23.32 | 0.48±0.04 | -4.49±0.83 |
| N-15 | 219.7±24.13 | 0.39±0.04 | -4.24±0.26 |
| N-16 | 99.53±5.53 | 0.92±0.04 | -1.78±4.08 |
| N-17 | 126.6±5.29 | 0.43±0 | 0.13±0.57 |
| N-18 | 134.2±10.57 | 0.47±0.04 | -10.87±8.73 |
| N-19 | 127±9.43 | 0.47±0.03 | 1.26±0.16 |
| N-20 | 170.43±7.34 | 0.46±0.02 | -1.2±0.94 |
| N-21 | 191.13±3.25 | 0.39±0.05 | -0.4±1.12 |
| N-22 | 115.87±31.14 | 0.73±0.16 | 0.49±1.33 |
| N-23 | 105.85±20.28 | 0.42±0.04 | -0.05±1.04 |
| N-24 | 182±43.8 | 0.4±0.02 | -7.92±4.94 |
| N-25 | 226.7±38.73 | 0.34±0.1 | 0.09±0.09 |
| N-26 | 194.27±12.13 | 0.37±0.03 | -7.16±2.31 |
| N-27 | 148.07±4.37 | 0.34±0.05 | -0.07±2.01 |
| N-28 | 250.17±38.65 | 0.49±0.11 | -16.4±9.53 |
| N-29 | 116.23±10.43 | 0.45±0.02 | -0.01±1.12 |
| N-30 | 259.57±12.85 | 0.42±0.11 | -19.3±5.56 |
| N-31 | 118.26±104.49 | 0.57±0.07 | -0.52±0.85 |
| N-32 | 234.8±35.57 | 0.37±0.07 | -1.64±0.62 |
| N-33 | 200.63±21.39 | 0.46±0.01 | -1.72±0.59 |
| N-34 | 142.63±19.28 | 0.47±0.17 | -2.27±1.29 |
| N-35 | 149.8±3.75 | 0.38±0.06 | -0.79±1.74 |
| N-36 | 750.13±237.2 | 0.55±0.06 | -4.63±2.54 |
| N-37 | 175.67±17.41 | 0.42±0.03 | -1.06±0.42 |
| N-38 | 177.77±7.83 | 0.39±0.02 | -17.3±2.63 |
| N-39 | 112.9±3.77 | 0.2±0.04 | -1.68±1.43 |
| N-40 | 168.1±5.75 | 0.41±0.01 | -33.17±5.58 |
| N-41 | 209.03±30.16 | 0.53±0 | -12.21±6.8 |
| N-42 | 236.73±17.81 | 0.49±0.11 | -21.97±5.01 |
| N-43 | 176.53±18.76 | 0.41±0.03 | -25.63±4.12 |
| N-44 | 725.73±770.99 | 0.68±0.29 | -4.6±0.63 |
| N-45 | 130.2±3.04 | 0.37±0.01 | -1.27±1.11 |
| N-46 | 252.43±137.66 | 0.47±0.04 | -34.5±9.16 |
| N-47 | 345.3±35.87 | 0.42±0.05 | -2.09±1.44 |
| N-48 | 132.6±11.62 | 0.82±0.03 | -4.04±5.28 |
| N-49 | 216.23±71.04 | 0.55±0.18 | -1.33±0.63 |
| N-50 | 112.47±11.97 | 0.44±0.01 | -16.25±9.21 |
| N-51 | 225.9±63.86 | 0.46±0.16 | -2.22±1.1 |
| N-52 | 143.63±11.38 | 0.47±0.06 | -15.84±10.22 |
| N-53 | 166.2±25.32 | 0.72±0.21 | -23.47±13.88 |
| N-54 | 147.93±12.59 | 0.42±0.05 | -13.87±3.31 |
| N-55 | 107.77±14.33 | 0.41±0.08 | -15.23±10.23 |
| N-56 | 175.8±5.99 | 0.51±0.04 | -8.29±10.71 |
| N-57 | 56.33±15.37 | 1±0 | -5.51±4.8 |
| N-58 | 132.07±10.53 | 0.54±0 | -2.88±2.32 |
| N-59 | 144.83±8.52 | 0.5±0.03 | -24.97±4.08 |
| N-60 | 98.04±6.22 | 0.43±0.02 | -13.23±8.44 |
| N-61 | 164.17±30.17 | 0.4±0.03 | -14.9±6.55 |
| N-62 | 141.87±41.3 | 0.67±0.35 | -2.42±2.87 |
| N-63 | 162.07±16.16 | 0.3±0.04 | -7.8±4.68 |
| N-64 | 142.87±1.27 | 0.41±0.02 | -15.97±2.77 |
| N-65 | 91.87±6.04 | 0.33±0.07 | -8.26±3.14 |
| N-66 | 317.37±87.4 | 0.41±0.04 | -8.46±5.59 |
| N-67 | 95.58±16.27 | 0.33±0.07 | -27.5±10.42 |
| N-68 | 253.23±16.18 | 0.53±0.1 | -27.53±9.2 |
| N-69 | 241.03±15.37 | 0.31±0.04 | -2.75±2.51 |
| N-70 | 142.4±11.52 | 0.21±0.05 | -4.31±3.54 |
| N-71 | 305.97±11.47 | 0.36±0.01 | -9.68±3.53 |
| N-72 | 129.2±5.67 | 0.69±0.18 | -15.91±10.73 |
| N-73 | 134.83±22.4 | 0.51±0.09 | 0.25±1.38 |
| N-74 | 106.37±6.17 | 0.31±0.02 | -12.98±5.44 |
| N-75 | 189.03±35.8 | 0.46±0.09 | -1.09±0.04 |
| N-76 | 176.8±25.42 | 0.28±0.05 | -0.52±1.51 |
| N-77 | 766.3±322.57 | 0.61±0.14 | -3.09±2.13 |
| N-78 | 187.13±7.75 | 0.49±0.1 | 0.05±2.33 |
| N-79 | 205.73±48.4 | 0.44±0.03 | -4.34±4.58 |
| N-80 | 108.99±12.21 | 0.41±0.07 | -1.06±1.89 |
| N-81 | 536.17±292.97 | 0.56±0.26 | -11.27±12 |
| N-82 | 133.53±6.54 | 0.33±0.06 | -1.78±0.78 |
| N-83 | 165.7±41.72 | 0.61±0.13 | -17.4±1.2 |
| N-84 | 143.47±4.13 | 0.4±0.01 | 0.98±0.19 |
| N-85 | 207.3±13.06 | 0.43±0.11 | -1.93±0.73 |
| N-86 | 337.9±49.44 | 0.59±0.24 | -7.38±6.44 |
| N-87 | 719.6±10.66 | 0.52±0.42 | -6.1±3.84 |
| N-88 | 150.27±21.79 | 0.4±0.06 | -4.16±4.61 |
| N-89 | 380.37±128.97 | 0.47±0.08 | -0.11±2.4 |
| N-90 | 191.8±18.69 | 0.3±0.14 | -4.54±2.52 |
| N-91 | 338.37±47.45 | 0.49±0.11 | -9.25±3.95 |
| N-92 | 589.07±9.75 | 0.31±0.35 | -8.39±2.1 |
| N-93 | 327.33±55.81 | 0.37±0.1 | -4.17±2.06 |
| N-94 | 66.24±43.24 | 1±0 | -2.31±0.89 |
| N-95 | 52.14±6.68 | 0.89±0.19 | -0.09±1.71 |
| N-96 | 114.27±15.62 | 0.46±0.02 | -0.88±2.38 |
| N-97 | 243.27±24.32 | 0.39±0.08 | -3.24±2.39 |
| N-98 | 266.07±23.25 | 0.33±0.05 | -19.6±5.78 |
| N-99 | 170.3±29.81 | 0.28±0.04 | -5.18±5.38 |
| N-100 | 56.42±8.17 | 0.91±0.09 | -21.83±12.62 |
| N-101 | 232.7±13.49 | 0.34±0.01 | -10.08±4.96 |
| N-102 | 146.67±3.07 | 0.31±0.04 | -19.4±5.37 |
| N-103 | 134.27±14.74 | 1±0 | -7.55±3.09 |
| N-104 | 51.52±19.63 | 0.67±0.31 | -3.55±3.3 |
| N-105 | 58.21±19.57 | 1±0 | -12.31±3.79 |
| N-106 | 100.59±6.34 | 0.89±0.03 | -18.33±5.85 |
| N-107 | 44.18±2.02 | 1±0 | -11.12±7.01 |
| N-108 | 81.9±14.64 | 0.85±0.02 | -2.86±4.51 |
| N-109 | 170.63±4.67 | 0.38±0.01 | -17.3±5.98 |
| N-110 | 61.79±10.03 | 0.97±0.05 | -15.23±14.55 |
| N-111 | 450.4±346.25 | 0.47±0.29 | -18.13±3.46 |
| N-112 | 74±12.11 | 0.98±0.03 | -0.37±1.59 |
| N-113 | 233.93±62.44 | 0.29±0.07 | -12.28±16.04 |
| N-114 | 238.93±15.54 | 0.39±0.01 | -16.77±5.88 |
| N-115 | 141.13±7.03 | 0.34±0.05 | -19.9±8.9 |
| N-116 | 820.37±608.57 | 0.62±0.33 | -13.7±3.38 |
| N-117 | 71.88±1.21 | 0.53±0.01 | -8.51±7.58 |
| N-118 | 169.63±6.01 | 0.3±0.06 | -7.86±1.57 |
| N-119 | 129.23±3.23 | 0.25±0.04 | -21.87±1.99 |
| N-120 | 76.67±20.85 | 0.31±0.16 | -0.12±3.56 |
| N-121 | 94.31±15.23 | 0.57±0.03 | -4.16±1.75 |
| N-122 | 158.57±26.78 | 0.45±0.04 | -9.24±1.3 |
| N-123 | 156.7±9.02 | 0.33±0.05 | -16.7±3.5 |
| N-124 | 77.13±4.53 | 0.51±0.03 | -0.22±2.29 |
| N-125 | 128.67±17.13 | 0.51±0.11 | -3.06±1.32 |
| N-126 | 87.35±5.83 | 0.63±0.21 | -7.84±2.14 |
| N-127 | 115.4±5.8 | 0.34±0.06 | -9.29±2.31 |
| N-128 | 88.3±11.01 | 0.52±0.02 | -18.03±4.34 |
| N-129 | 371.67±103.52 | 0.51±0.18 | -14.23±7.73 |
| N-130 | 150.2±9.24 | 0.4±0.06 | -15.93±3.46 |
| N-131 | 347.3±175.53 | 0.46±0.03 | -1.76±0.78 |
| N-132 | 170.4±9.28 | 0.26±0.02 | -22.9±6.15 |
| N-133 | 70.5±61.7 | 0.25±0.08 | -0.96±5.64 |
| N-134 | 206.57±44.25 | 0.5±0.27 | -5.64±8.2 |
| N-135 | 266±11.46 | 0.29±0.01 | -18.6±6.6 |
| N-136 | 121.2±6.92 | 0.42±0.11 | -6.19±4.49 |
| N-137 | 131±26.05 | 0.68±0.34 | -1.64±3.62 |
| N-138 | 89.77±3.64 | 0.4±0.09 | -5.51±1.04 |
| N-139 | 134.17±3.57 | 0.25±0.01 | -3.65±2.17 |
| N-140 | 87.7±14.66 | 0.43±0.08 | -6.57±3.08 |
| N-141 | 286.83±12.25 | 0.4±0.04 | -13.82±3.93 |
| N-142 | 159.17±24.13 | 0.41±0.07 | -2.43±1.14 |
| N-143 | 263.3±21.22 | 0.32±0.01 | -16.67±9.63 |
| N-144 | 119.27±1.99 | 0.32±0.05 | -14.55±6.84 |
| N-145 | 80.51±11.82 | 0.45±0.02 | 0.38±1.05 |
| N-146 | 234.23±24.55 | 0.43±0.09 | -9.84±9.76 |
| N-147 | 122.9±7.17 | 0.25±0.02 | -1.97±1.34 |
| N-148 | 212.87±48.94 | 0.55±0.07 | -11.62±9.71 |
| N-149 | 135.7±10.82 | 0.26±0.04 | -5.37±4.8 |
| N-150 | 163.33±23.04 | 0.72±0.22 | -8.8±8.01 |
| N-151 | 393±29.47 | 0.42±0.01 | -2.69±2.63 |
| N-152 | 344.5±209.09 | 0.47±0.15 | -0.5±0.46 |
| N-153 | 210.57±71.45 | 0.29±0.04 | -5.43±4.29 |
| N-154 | 172.4±17.87 | 0.24±0.06 | -25.2±2.49 |
| N-155 | 147.47±10.76 | 0.32±0.14 | -6.68±3.4 |
| N-156 | 226.17±92.11 | 0.31±0.11 | -0.56±0.75 |
| N-157 | 162.43±23.44 | 0.41±0.03 | -11.85±3.47 |
| N-158 | 80.1±92.55 | 0.33±0.05 | 0.23±0.73 |
| N-159 | 142.97±12.72 | 0.15±0.04 | -0.36±0.41 |
| N-160 | 413.9±76.36 | 0.41±0.04 | -0.94±0.41 |
| N-161 | 229.27±9 | 0.25±0.03 | -0.97±0.34 |
| N-162 | 113.8±12.24 | 0.21±0.06 | -17.1±1.57 |
| N-163 | 174.97±63.72 | 0.57±0.3 | 0.35±0.65 |
| N-164 | 272.37±78.21 | 0.27±0.07 | -11.73±11.88 |
| N-165 | 117.53±20.58 | 0.63±0.08 | -25.53±5.49 |
| N-166 | 179.97±40.88 | 0.47±0.09 | -22.97±4.55 |
| N-167 | 872.87±95.21 | 0.87±0.13 | -26.87±3.69 |
| N-168 | 133.33±3.07 | 0.22±0.02 | -0.65±1.51 |
| N-169 | 235.33±13.17 | 0.53±0.05 | -25.23±2.04 |
| N-170 | 103.33±2.75 | 0.34±0.02 | -16.23±4.18 |
| N-171 | 140.2±31.61 | 0.36±0.13 | -25.97±1.76 |
| N-172 | 156.87±27.69 | 0.72±0.3 | -2.95±1.89 |
| N-173 | 65.66±2.59 | 0.57±0.02 | -22.27±9.68 |
| N-174 | 77.65±34.61 | 0.87±0.22 | -6.3±8.57 |
| N-175 | 61.69±14.65 | 1±0 | -14.83±10.13 |
| N-176 | 134.3±29.95 | 0.67±0.35 | -2.37±0.85 |
| N-177 | 48.54±5.84 | 1±0 | -9.7±14.03 |
| N-178 | 157.83±55.97 | 0.68±0.19 | -12.89±6.1 |
| N-179 | 116.59±19.87 | 0.74±0.09 | -12.1±5.9 |
| N-180 | 171.4±34.38 | 0.52±0.19 | -1.94±0.82 |
| N-181 | 149.76±122.02 | 0.67±0.28 | -0.94±0.82 |
| N-182 | 206.03±29.57 | 0.79±0.19 | -0.83±2.26 |
| N-183 | 139.16±48.82 | 0.9±0.17 | -6.43±1.94 |
| N-184 | 71.04±7.96 | 0.55±0.01 | -0.52±0.58 |
| N-185 | 135.5±3.5 | 0.27±0.01 | -7.35±3.51 |
| N-186 | 233.5±8.51 | 0.48±0.02 | -9.8±1.66 |
| N-187 | 66.42±15.29 | 0.93±0.12 | -5.07±6.78 |
| N-188 | 85.36±34.12 | 0.65±0.31 | -1.41±1.96 |
| N-189 | 182.87±47.09 | 0.3±0.12 | -0.07±0.64 |
| N-190 | 149±27.83 | 0.45±0.12 | -2.35±3.05 |
| N-191 | 204.7±25.03 | 0.27±0.06 | -14.68±11.54 |
| N-192 | 81.34±6.2 | 0.59±0.01 | -4.21±1.85 |
| N-193 | 163.72±184.68 | 1±0 | 0±1.63 |
| N-194 | 146.8±8.58 | 0.34±0.01 | -12.63±5.96 |
| N-195 | 220.9±31.39 | 0.4±0.08 | -3.81±2.14 |
| N-196 | 204.37±34.9 | 0.23±0.05 | -6.17±1.28 |
| N-197 | 80.15±5.87 | 0.65±0.05 | -4.26±2.42 |
| N-198 | 150.43±4.79 | 0.34±0.05 | -1.1±0.95 |
| N-199 | 193.97±124.63 | 0.66±0.13 | -5.64±0.55 |
| N-200 | 162.17±15.87 | 0.45±0.13 | -9.18±5.16 |
| N-201 | 159.2±7.08 | 0.25±0.06 | -11.64±2.46 |
| N-202 | 566.83±28.92 | 0.1±0.07 | -22±1.75 |
| N-203 | 154.67±3.15 | 0.22±0.01 | -20.83±2.86 |
| N-204 | 200.23±48.36 | 0.51±0.09 | -3.56±5.02 |
| N-205 | 150.6±10.22 | 0.41±0.03 | -19.63±2.9 |
| N-206 | 117.7±2.48 | 0.44±0.03 | -3.64±1.92 |
| N-207 | 54.06±9.88 | 0.66±0.13 | -0.48±2.47 |
| N-208 | 230±6.77 | 0.51±0.13 | -0.92±1.77 |
| N-209 | 129.8±15.44 | 0.7±0.04 | -12.24±4.86 |
| N-210 | 66.02±4.12 | 0.49±0.02 | -1.41±2.77 |
| N-211 | 156.67±2.03 | 0.39±0.04 | -24.27±0.71 |
| N-212 | 208.4±38.07 | 0.39±0.03 | -0.24±1.24 |
| N-213 | 121.11±22.57 | 0.49±0.02 | -10.47±4.26 |
| N-214 | 80.84±13.03 | 0.59±0.06 | -1.23±3.32 |
| N-215 | 268.13±17.07 | 0.39±0.07 | -24.57±6.09 |
| N-216 | 516.53±307.61 | 0.48±0.17 | 0.14±0.66 |
| N-217 | 507.37±319.4 | 0.53±0.26 | -22.1±4 |
| N-218 | 96.97±10.01 | 0.47±0.05 | -1.28±0.95 |
| N-219 | 101.43±8.37 | 0.42±0.03 | -7.15±6.17 |
| N-220 | 410.13±36.72 | 0.37±0.06 | -1.37±1.27 |
| N-221 | 112.07±4.3 | 0.27±0 | -1.93±1.36 |
| N-222 | 217.43±36.62 | 0.42±0.19 | -1.81±2.98 |
| N-223 | 69.37±19.09 | 0.55±0.28 | -14.1±2.05 |
| N-224 | 149.87±8.14 | 0.41±0.02 | -9.95±3.02 |
| N-225 | 150.83±15.35 | 0.31±0.08 | -24.9±5.63 |
| N-226 | 81.44±28.9 | 1±0 | -15.02±6.43 |
| N-227 | 65.29±4.26 | 0.6±0.02 | -2.69±3.04 |
| N-228 | 23.18±8.58 | 0.52±0.11 | -1.15±2.73 |
| N-229 | 87.61±2.27 | 1±0 | -14.14±6.49 |
| N-230 | 111.73±1.3 | 0.25±0.01 | -13.86±5.3 |
| N-231 | 215.27±6.8 | 0.47±0.09 | -41.1±6.75 |
| N-232 | 287.17±16.69 | 0.97±0.05 | -17.7±2.35 |
| N-233 | 181.03±16.43 | 0.27±0.02 | -0.09±2.01 |
| N-234 | 166.3±59.01 | 0.26±0.03 | -16.79±15.14 |
| N-235 | 131.7±5.36 | 0.29±0.03 | -7.67±7.15 |
| N-236 | 553.3±207.84 | 0.93±0.12 | -18.63±0.84 |
| N-237 | 3488.33±407.51 | 0.59±0.35 | -2.83±2.17 |
| N-238 | 57.29±19.54 | 0.96±0.06 | -22.03±11.53 |
| N-239 | 151.03±95.96 | 0.25±0.03 | -1.89±2.18 |
| N-240 | 53.31±3.67 | 0.35±0.24 | -22.97±16.51 |
| N-241 | 110.19±85.66 | 0.22±0.03 | -1.78±0.42 |
| N-242 | 483.3±32.24 | 0.19±0.03 | -5.65±1.77 |
| N-243 | 93.96±2.4 | 0.62±0.05 | -3.09±1.06 |
| N-244 | 54.04±13.81 | 0.97±0.05 | -5.24±7.98 |
| N-245 | 91.13±2.48 | 0.43±0.02 | -1.63±3.05 |
| N-246 | 114.53±5.31 | 0.47±0.04 | -18.2±2.85 |
| N-247 | 128.83±1.33 | 0.53±0 | -2.84±1.79 |
| N-248 | 48.19±56.23 | 0.52±0.28 | -0.83±1.65 |

**Table S8.** Cytotoxicity screening assay in Lewis cells

| Sample | Size（d.nm） | | PDI | Zeta Potential（mV） | IC50（mg/mL） | Ingredients |
| --- | --- | --- | --- | --- | --- | --- |
| N-1 | 146.66±21.59 | | 0.282±0.039 | -3.35±1.67 | 0.023 | Peimine, Peiminine, Peimisine, Ginsenoside Re, Ginsenoside Ro, Rosmarinic acid, Glycyrrhizic acid, Ganoderic acid H, Citric acid |
| N-4 | 242.43±31.06 | 0.14±0.07 | | -7.37±0.61 | 0.039 | Peimine, Peiminine, Ginsenoside Re, Ginsenoside Ro, Rosmarinic acid, Glycyrrhizic acid, Ganoderic acid H, Citric acid |
| N-7 | 173.73±33.19 | 0.23±0.06 | | -17.33±1.51 | 0.03 | Peimine, Peiminine, Peimisine, Ginsenoside Re, Ginsenoside Ro, Glycyrrhizic acid, Ganoderic acid H, Citric acid |
| N-8 | 155.83±5.52 | 0.35±0.01 | | -30.2±3.44 | 0.013 | Peimine, Peiminine, Peimisine, Ginsenoside Re, Ginsenoside Ro, Rosmarinic acid, Ganoderic acid H, Citric acid |
| N-24 | 182±43.8 | 0.4±0.02 | | -7.92±4.94 | 0.00002056 | Peimine, Peiminine, Ginsenoside Ro, Rosmarinic acid, Glycyrrhizic acid, Ganoderic acid H, Citric acid |
| N-39 | 112.9±3.77 | 0.2±0.04 | | -1.68±1.43 | 0.0000129 | Ginsenoside Re, Ginsenoside Ro, rosmarinic acid, glycyrrhizic acid, ganoderic acid H, citric acid |
| N-45 | 130.2±3.04 | 0.37±0.01 | | -1.27±1.11 | 0.001057 | Peimisine, ginsenoside Ro, rosmarinic acid, glycyrrhizic acid, ganoderic acid H, citric acid |
| N-65 | 91.87±6.04 | 0.33±0.07 | | -8.26±3.14 | 0.0007253 | Peimisine, ginsenoside Re, ginsenoside Ro, glycyrrhizic acid, ganoderic acid H, citric acid |
| N-67 | 95.58±16.27 | 0.33±0.07 | | -27.5±10.42 | 0.04653 | Peimisine, ginsenoside Re, ginsenoside Ro, glycyrrhizic acid, citric acid |
| N-74 | 106.37±6.17 | 0.31±0.02 | | -12.98±5.44 | 0.0767 | Peimisine, ginsenoside Re, ginsenoside Ro, rosmarinic acid, glycyrrhizic acid, ganoderic acid H |
| N-97 | 243.27±24.32 | 0.39±0.08 | | -3.24±2.39 | 0.01832 | Peimisine, ginsenoside Ro, ganoderic acid H, citric acid |
| N-98 | 266.07±23.25 | 0.33±0.05 | | -19.6±5.78 | 0.03357 | Peimisine, ginsenoside Re, ginsenoside Ro, citric acid |
| N-115 | 141.13±7.03 | 0.34±0.05 | | -19.9±8.9 | 0.01681 | Peimisine, ginsenoside Re, ginsenoside Ro, citric acid Saponin Ro, rosmarinic acid, citric acid |
| N-119 | 129.23±3.23 | 0.25±0.04 | | -21.87±1.99 | 0.07177 | Peiminine, ginsenoside Re, ginsenoside Ro, glycyrrhizic acid, ganoderic acid H |
| N-127 | 115.4±5.8 | 0.34±0.06 | | -9.29±2.31 | 0.06069 | Peiminine, Peimisine, ginsenoside Ro, glycyrrhizic acid, ganoderic acid H |
| N-132 | 170.4±9.28 | 0.26±0.02 | | -22.9±6.15 | 0.1113 | Peimine, Peimisine, ginsenoside Re, ginsenoside Ro, citric acid |
| N-139 | 134.17±3.57 | 0.25±0.01 | | -3.65±2.17 | 0.07972 | Peimine, ginsenoside Ro, rosmarinic acid, glycyrrhizic acid, ganoderic acid H |
| N-147 | 122.9±7.17 | 0.25±0.02 | | -1.97±1.34 | 0.1108 | Peimine, Peimisine, ginsenoside Ro, glycyrrhizic acid, ganoderic acid H |
| N-149 | 135.7±10.82 | 0.26±0.04 | | -5.37±4.8 | 0.1209 | Peimine, Peimisine, ginsenoside Re, ginsenoside Ro, glycyrrhizic acid |
| N-154 | 172.4±17.87 | 0.24±0.06 | | -25.2±2.49 | 0.04789 | Peimine, Peiminine, ginsenoside Ro, rosmarinic acid, glycyrrhizic acid |
| N-159 | 142.97±12.72 | 0.15±0.04 | | -0.36±0.41 | 0.07795 | Peimine, Peiminine, ginsenoside Re , ginsenoside Ro, glycyrrhizic acid |
| N-162 | 113.8±12.24 | 0.21±0.06 | | -17.1±1.57 | 0.01977 | Peimine, Peiminine, Peimisine, ginsenoside Ro, glycyrrhizic acid |
| N-168 | 133.33±3.07 | 0.22±0.02 | | -0.65±1.51 | 0.006694 | Peimisine, ginsenoside Re, ginsenoside Ro, rosmarinic acid |
| N-185 | 135.5±3.5 | 0.27±0.01 | | -7.35±3.51 | 0.01084 | Peiminine, Peimisine, ginsenoside Ro, citric acid |
| N-196 | 204.37±34.9 | 0.23±0.05 | | -6.17±1.28 | 0.01268 | Peiminine, ginsenoside Ro, rosmarinic acid, glycyrrhizic acid |
| N-201 | 159.2±7.08 | 0.25±0.06 | | -11.64±2.46 | 0.0135 | Peimine, Peimisine, ginsenoside Ro, citric acid |
| N-203 | 154.67±3.15 | 0.22±0.01 | | -20.83±2.86 | 0.02177 | Peimine, ginsenoside Re, ginsenoside Ro, citric acid |
| N-205 | 150.6±10.22 | 0.41±0.03 | | -19.63±2.9 | 0.02575 | Peimine, ginsenoside Ro, rosmarinic acid, citric acid |
| N-221 | 112.07±4.3 | 0.27±0 | | -1.93±1.36 | 0.01483 | Peiminine, ginsenoside Ro, citric acid |
| N-230 | 111.73±1.3 | 0.25±0.01 | | -13.86±5.3 | 0.00228 | Peimisine, ginsenoside Ro, rosmarinic acid |
| N-235 | 131.7±5.36 | 0.29±0.03 | | -7.67±7.15 | 0.003292 | Ginsenoside Re, ginsenoside Ro, rosmarinic acid |

**
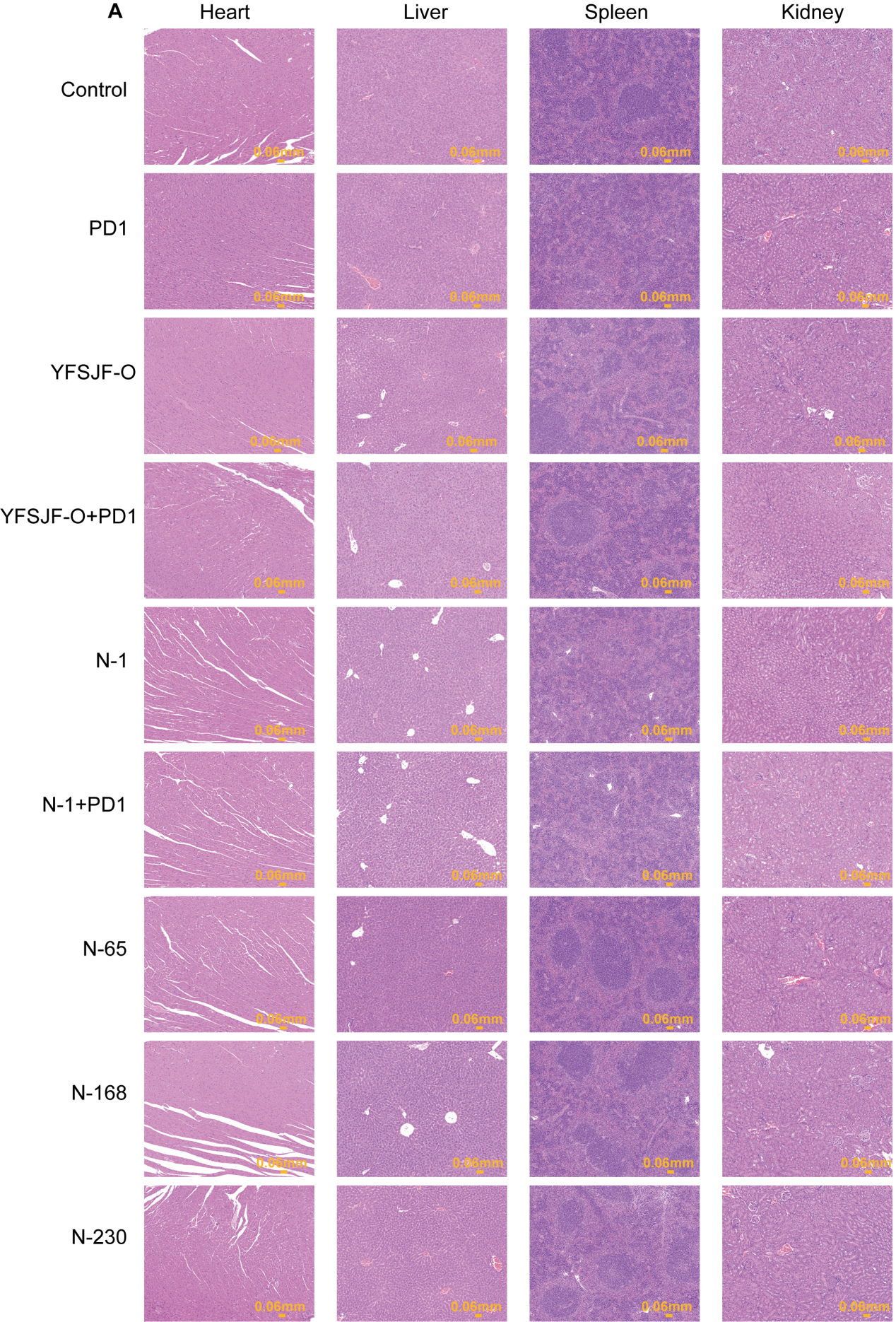
Fig S6.** A HE staining of heart, liver, spleen and kidney of 9 groups, including Control, PD1, YFSJF-O, YFSJF-O+PD1, N-1, N-1+PD1, N-65, N-168 and N-230.


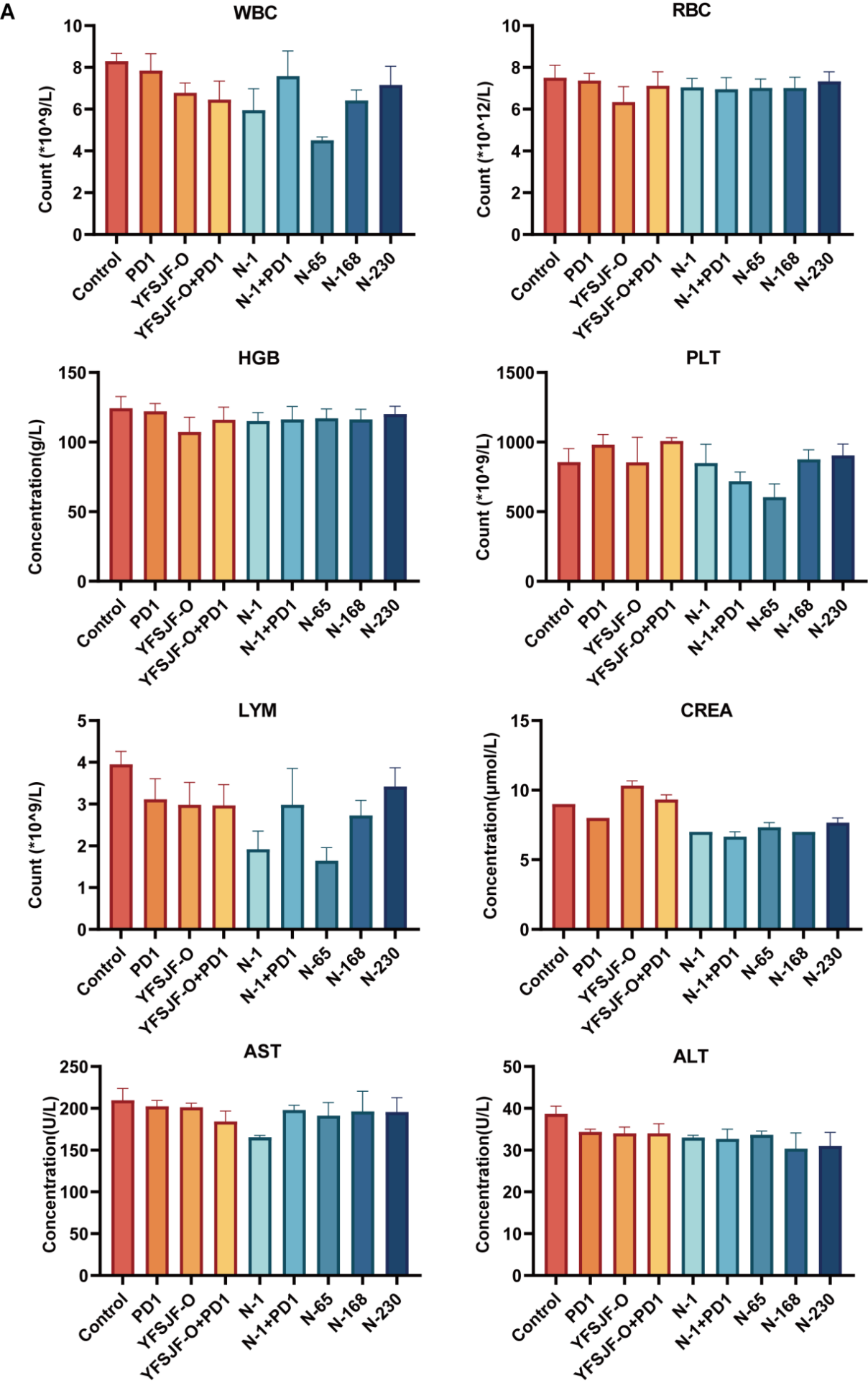
**Fig S7.** A is the blood test values of red blood cells, white blood cells, hemoglobin, platelets, lymphocytes, ALT, AST, and creatinine of the 9 groups.

**References**

[1] M Gao YC, H Fan SC, H Wang WN, et al. Transgenerational effects on the gene transcriptome of chicken liver. Animal Advances. 2024, 1(1).

[2] AM Bolger ML, Usadel B. Trimmomatic: a flexible trimmer for Illumina sequence data. Bioinformatics. 2014, 30(15): 2114-2120.

[3] Thakur V. RNA-Seq Data Analysis for Differential Gene Expression Using HISAT2–StringTie–Ballgown Pipeline. Transcriptome Data Analysis. 2024: 101-113.

[4] K Okonechnikov AC, García-Alcalde F. Qualimap 2: advanced multi-sample quality control for high-throughput sequencing data. Bioinformatics. 2016, 32.

[5] Y Du QH, Arisdakessian C. Evaluation of STAR and Kallisto on single cell RNA-Seq data alignment. G3: Genes, Genomes. 2020, 10(5): 1775-1783.

[6] N Wang YL, S Han YZ, J Yang ZY, et al. CFViSA: a comprehensive and free platform for visualization and statistics in omics-data. Comput Biol Med. 2024, 171: 108206.

[7] Y Ke HJ, Z Ping WY, X Na YJ, et al. The progressive application of single-cell RNA sequencing technology in cardiovascular diseases. BIOMEDICINE & PHARMACOTHERAPY. 2022, 154: 113604.

[8] B Sarker MMR, MH Alamin MAI, Mollah M. Boosting edgeR (Robust) by dealing with missing observations and gene-specific outliers in RNA-Seq profiles and its application to explore biomarker genes for diagnosis and therapies of ovarian cancer. Genomics. 2024, 116(3): 110834.

[9] DV Klopfenstein LZ, BS Pedersen FR. GOATOOLS: A Python library for Gene Ontology analyses. Sci Rep. 2018, 8(1): 10872.

[10] D Bu HL, P Huo ZW, S Zhang ZH, et al. KOBAS-i: intelligent prioritization and exploratory visualization of biological functions for gene enrichment analysis. Nucleic Acids Res. 2021,49(W1): W317-W325.

[11] H Yan SW, H Liu HM, Zhu S. GORetriever: reranking protein-description-based GO candidates by literature-driven deep information retrieval for protein function annotation. Bioinformatics. 2024, 40(Supplement_2): ii53-ii61.

[12] Y Lyu XL, S Gao JL, J Li SZ, et al. Cpd861 Targeting BCL2 to Alleviate Hepatic Fibrosis: Network Pharmacology, Mendelian Randomization, and Molecular Docking Mechanisms. Curr Pharm Des. 2024, 30(41): 3291-3310.

[13] D Tang MC, X Huang GZ, L Zeng GZ, et al. SRplot: A free online platform for data visualization and graphing. PLoS ONE. 2023, 18, e0294236.
